# Open-source, low-cost, in-situ turbidity sensor for river network monitoring

**DOI:** 10.1038/s41598-022-14228-4

**Published:** 2022-06-20

**Authors:** Jessica Droujko, Peter Molnar

**Affiliations:** Institute of Environmental Engineering, ETH Zurich, Zurich, 8093 Switzerland

**Keywords:** Environmental sciences, Hydrology, Engineering

## Abstract

Fine sediment transport in rivers is important for catchment nutrient fluxes, global biogeochemical cycles, water quality and pollution in riverine, coastal and marine ecosystems. Monitoring of suspended sediment in rivers with current sensors is challenging and expensive and most monitoring setups are restricted to few single site measurements. To better understand the spatial heterogeneity of fine sediment sources and transport in river networks there is a need for new smart water turbidity sensing that is multi-site, accurate and affordable. In this work, we have created such a sensor, which detects scattered light from an LED source using two detectors in a control volume, and can be placed in a river. We compare several replicates of our sensor to different commercial turbidity probes in a mixing tank experiment using two sediment types over a wide range of typical concentrations observed in rivers. Our results show that we can achieve precise and reproducible turbidity measurements in the 0–4000 NTU or 0–16g/L range. Our sensor can also be used directly as a suspended sediment sensor and bypass an unnecessary calibration to Formazin. The developed turbidity sensor is much cheaper than existing options of comparable quality and is especially intended for distributed sensing across river networks.

## Introduction

Fine sediment production and transport is an important process at the global scale, affecting riverine, coastal and marine ecosystems^1–3^. Present-day fine sediment export from the land surface to global oceans by large rivers is estimated at about 15.5–18.5 Gt per year^4,5^ and this is about half of the estimated global annual soil erosion from the land surface^6^. However, it remains very difficult to estimate suspended sediment yields in rivers because of the high variability in along-stream suspended sediment concentrations (SSC) and inadequate monitoring thereof.

The primary method to determine SSC is gravimetric analysis of bottle samples taken at river cross-sections in regular or irregular intervals. This method is reliable but has many disadvantages such as being discontinuous (poor temporal resolution), inefficient and costly (high effort for collection/transport/analysis of the samples, long processing times). Sediment budgets for river basins are usually derived from these direct measurements of SSC^7,8^. Continuous SSC data at high temporal resolutions can be obtained by dedicated in-situ sensors which measure turbidity (T), and by calibrating a relation between SSC and T. In river cross-sections where measurements of water discharge (Q) are also made, sediment yield (QS) is then computed as QS = SSC*Q. High resolution estimates of SSC can be used to quantify human effects on sediment production, for example the effects of dam construction and erosion control^9,10^, the natural erosion gradients over entire mountain ranges^11^, the role of sampling in global erosion rates^12^, and many others. Measurements of SSC at a basin outlet can give us a basin-integrated picture of possible hydroclimatically-driven sources of sediment, like rainfall erosion, snowmelt hillslope erosion, glacier ice melt erosion, even hydropower storage in dams^13^. These measurements of SSC are also important for understanding the impact of hydroclimatic forcing on activating sediment sources and transport dynamics, and physically-based modelling thereof^14–18^.

High temporal resolution monitoring using in-situ turbidity sensors at, for example, glacier streams is useful for identifying the time-dependent sediment export rates connected to the development and evolution of subglacial channels and the possible contributions of proglacial sediment sources^19,20^. Together with grain size measurements, highly resolved SSC data can be used for the detailed hydraulic modelling of glacially-derived sediment transport by meltwater drainage in subglacial streams^21^. Hillslope source connectivity to the fluvial network in proglacial areas is also an important modulator of time-dependent sediment production^14,22^. Such process understanding requires a spatial perspective on sediment pathways of production and storage within the catchment which cannot be achieved by single-site measurements. Additionally, the main deficiency of point river measurements by devoted suspended sediment monitoring sensors is that they are expensive (e.g. state-of-the-art turbidity sensor by Campbell is about 6000, In-Situ is 7000), making widespread deployment at many sites along a river system to quantify spatial variability next to impossible. Nevertheless, this is currently the state-of-the-art measurement in both small and large river systems.

An alternative to ground point measurements is remote sensing. The spatial distribution of SSC can be obtained using satellite imagery which is based on the reflectance of water surface as it is affected by a range of parameters (chlorophyll, suspended sediment, dissolved organic matter, etc.). Dissolved and suspended sediment concentrations together with gross biological activity affect the intrinsic colour of natural waters^23,24^, which makes optical satellite remote sensing of oceans, coastal areas, large lakes/rivers possible. When calibrated with ground measurements, such satellite data can be very useful for SSC estimates^25,26^ and can give a range of additional water quality parameters^27^ at large scales but not with high temporal resolutions (repeatability given by satellite overpasses) and with poor point accuracy. In rivers, satellite-based analyses are only possible if the spatial footprint is sufficiently large. For example, in the Amazon SSC variations with satellite imagery were shown to follow overbank flow, and could be explained by resuspension of sediments in depression lakes^28^ only because the river is very wide.

Optical sensing of river turbidity, and other water quality indicators, is also possible with terrestrial photography^29,30^. Optical sensing of turbidity by mobile phone cameras^31^ is an application that has broad appeal for some ground applications. However, all ground-based and UAV optical sensing methods are limited by cost, poor temporal resolution, and are strongly affected by many environmental constraints (light, good optical transmission, visibility, etc.), which make them currently not very suitable for regular long-term monitoring of SSC in rivers.

We argue that a new type of sediment monitoring is necessary to demonstrate the many physical connections between hydrology, river processes, and sediment fluxes. The connections between sediment source activation and transport on hillslopes, cultivated fields, vegetated surfaces, subglacial channel networks, in streams, large rivers, and deltas, all require sediment monitoring at high spatial (from source to sink) and temporal (activation timescale) resolutions that are not fully guaranteed by any of the standard approaches in economically-effective ways. This new data is needed for developing an understanding of sediment storages and budgets in Alpine basins^32–34^, revealing intricate details of sediment connectivity in such fluvial systems^14,22^, and for calibrating physically-based hydrological-sediment transport models^15,17,35,36^. For this reason, we propose here an affordable turbidity sensor that can be used to create a distributed suspended sediment monitoring network.

### State-of-the-art in low-cost turbidity sensing

Several novel turbidity sensors have been documented in peer-reviewed literature. Gillett^37^ investigated the use of low-cost, commercially-available appliance turbidity sensors (such as those found in washing machines and dishwashers). These sensors work on the principle of light attenuation, where the light detector is placed 180$^o$ from the incident light. Gillet^37^ found that these analog attenuation sensors could not achieve a sufficiently high resolution to monitor small changes in turbidity. Trevathan^38^ recalibrated an appliance sensor (DF Robot SEN0189 Gravity - also attenuation style) and built a waterproof housing for field deployments. However, they did not report on the accuracy of the sensor and were not able to calibrate below 100 Nephelometric Turbidity Units (NTU) (although their test measurements were between 0-20 NTU). Additionally, the data obtained by their sensor was influenced by ambient stray light and they were not able to overcome this problem.

To improve the accuracy of an attenuation-style sensor in the 0-100 NTU range, Lambrou^39^ and Wang^40^ equipped these attenuation sensors with a detector at $90^o$ to the incident beam. This method provides good stability, linearity, sensitivity, low stray light, and increases the NTU measurement range (when incorporating the backscatter detector)^41^. The use of several detectors at different angles allows the partial cancellation of errors due to wavelength absorption in samples^42^. However, Lambrou’s^39^ sensor was only tested from 0-100 NTU and did not report on how the 0.1 NTU resolution was obtained. Additionally, Wang^40^ only tested their sensor within the 0-1000 NTU range. Kelley^43^ created a low-cost, hand-held turbidity meter meant for investigating drinking water quality in low-income communities around the world. This sensor was calibrated with and tested against a commercial turbidity sensor but only in the 0-1000 NTU range. Additionally, using commercial turbidity sensors to calibrate is not advisable since their measurements have strong differences in reported values among the various sensors^44^. Kitchener^45^ conducted a sediment settling experiment by constructing a modular PVC ring that could hold light detectors at several angles ($0^o$, $10^o$, $20^o$, $90^o$, $160^o$) to the illuminating LED. They reported their results in SI units of radiant intensity (mW/sr) instead of using formazin and obtaining units of NTU. Their device did however cost around 340GBP and cannot be deployed for environmental applications.

In addition to testing the appliance sensors, Gillett^37^ created a flow-through sensor meant to be attached to a PVC and continuously monitor turbidity for 64. However, due to the nature of their design (large PVC diameter), they used a very powerful ambient LED and calibrated their sensor under dark-room conditions. This sensor can be used in a pump-test setup, as they’ve shown, but is unsuitable for environmental applications where ambient stray light will interfere with the measurement. Jiang^46^ created a turbidity sensor for deep-sea applications, reaching depths of over 3400 meters. The sensor was based on a backscatter principle (the detector is positioned at < $45^o$ relative to the incident LED beam) but is only suitable in the 0-20 FNU range (FNU and NTU are interchangeable units of turbidity). Additionally, the sensor is claimed to cost under 40 however, the bill of materials does not include sensor housing costs and all of the components used.

### New sensor design

In this paper, we propose an open-source and low-cost turbidity sensor that can be used for in-situ river network deployment. Based on the review above and on the work carried out in the Supplementary Methods—First prototype section, we have designed a sensor with the following features. (1) The sensor has one light source and two detectors at different angles to each other for partial cancellation of errors^42^. (2) It takes the difference between two measurements (LED off and on) to reduce ambient light effects. (3) It covers a large suspended sediment range 0–4000 NTU or 0-16 g/L concentration. (4) The sensor is low-cost and open-source so it can be built by users. (5) The sensor can be installed in a river system for in-situ measurements.

**Figure 1:**
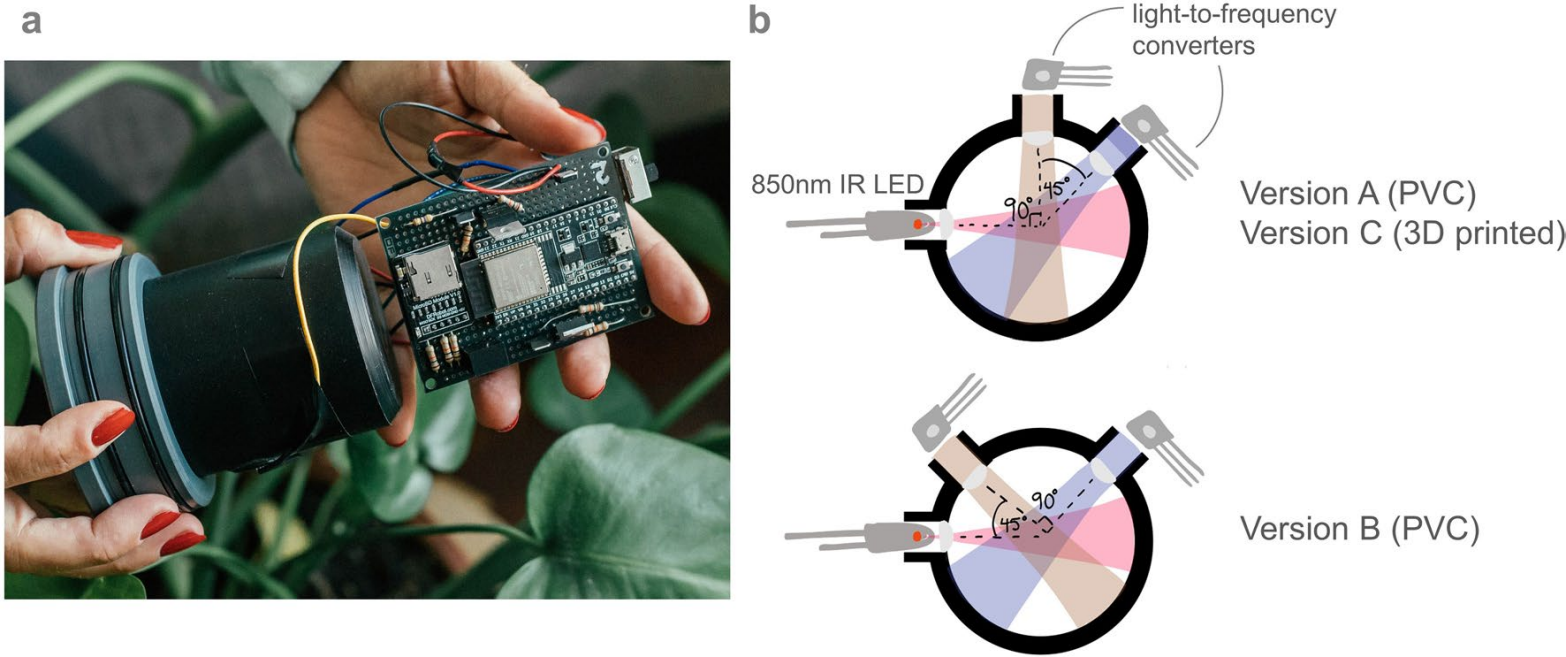
Our open-source sensor. (**a**) the sensor without the waterproof housing (see Methods for the sensor with housing), the sensing head is in black machined PVC, the circuit board is also shown, (**b**) operating principle behind our open-source sensors, an LED illuminated the sample in the cavity and the quantity of scattered light is measured by the detectors in Hz, three different versions are depicted: versions A and B are made from machined PVC whereas version C is 3D printed, versions A and C have detectors at $90^o$ and $135^o$ relative to the LED and version B has detectors at $45^o$ and $135^o$.

We built three different versions of our open-source sensor, which can be seen in Fig. 1. The operating principle of all three versions are the same, the differences lie in the construction of the devices and in the detector angles within the control volume. Versions A and B are made from machined PVC whereas version C is 3D printed from PLA. Versions A and C have detectors at $90^o$ and $135^o$ relative to the LED and version B has detectors at $45^o$ and $135^o$. We built 2–3 replicates of each version giving a total of eight open-source sensors to test in this work. For more details on versions A, B, and C, see Methods and Supplementary Table S4.

## Results and discussion

### Comparison NTU-SSC between open-source sensor and commercial sensors

We tested sensor versions A–B–C against three Endress+Hauser (E &H) sensors (two are model CUS51D and one is CUS52D) in a mixing tank setup with two different sediment types: Feldspar ($d_{50}=30\, \mu m$) and sediment collected from the Fieschertal channel ($d_{50}=90\, \mu m$)^44^.

**Figure 2:**
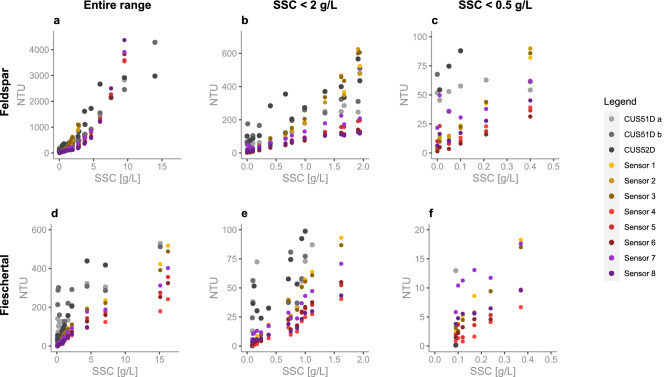
Mean NTU vs. SSC comparison in Feldspar sediment (**a**–**c**) and Fieschertal sediment (**d**–**f**) for the full measurement range (a and d), from 0 to 2 g/L (**b** and **e**), and from 0 to 0.5 g/L (**c** and **f**). The three E &H sensors are plotted in grey gradient (first CUS51D in light-grey, second CUS51D in mid-grey, CUS52D in dark grey). Version A sensors are in yellow gradient (Sensor 1 in bright yellow, Sensor 2 in mid-yellow, Sensor 3 in dark yellow). Version B sensors are in red gradient (Sensor 4 in bright red, Sensor 5 in mid-red, Sensor 6 in dark-red). Version C sensors are in purple gradient (Sensor 7 in bright purple, Sensor 8 in dark purple). At higher SSC one of the 3D printed (version C) sensors failed.

Figure 2 shows the eight open-source sensor results overlayed with the three E &H results for Feldspar (Fig. 2a–c) and Fieschertal (Fig. 2d–f) sediment in three different ranges. All eight open-source sensors are plotted with version A in yellow gradient, version B in red gradient, and version C in purple gradient. Every point is a mean which was computed using all of the values, at every SSC level, recorded over the length of the experiment (30min per every SSC level in Feldspar and 15min per every SSC level in Fieschertal - see Methods for more details).

Our open-source sensors provide very similar measurements between the replicates of individual sensor versions for the full 0-16 g/L range, down to 0.25 g/L (Fig. 2b–c and e–f). As expected, at these very low concentrations the sensor replicates start to disagree. For example, the three version B sensors (red gradient) show that all measurements are repeatable down to 0.05 g/L (in Feldspar) and 0.25 g/L (in Fieschertal). Our version C sensors (purple gradient) follow a similar trend down to 0.1 g/L (in Feldspar) and 0.25 g/L (in Fieschertal) but with an offset in the NTU values between the two version C sensors therefore, creating less “repeatable” results (NTU comparison between sensors is more difficult). One possible explanation could be that these sensors were 3D printed with a hobby-printer, and are therefore less precise, than the machined sensors. Another possible explanation for the offset is the sensor placement around the cylindrical tank (see Fig.10). We consider version A and B sensors to be suitable for future testing and possibly for distributed applications. Alternatively, the version C sensors can be used for the monitoring of trends, but we have lower confidence in the absolute values.

In contrast, the E &H sensors do not provide similar measurements for all three sensors or even when comparing two sensors of the same model (CUS51D) for the entire measurement range. Both CUS51D sensors do provide similar measurements in both Feldspar and Fieschertal but only above 2g/L (Fig. 2a and d), meaning that these two sensors give “repeatable” results (turbidities could be compared at two sites along a river network with these sensors). In general, the CUS51Ds’ measurements cannot be compared to the CUS52D’s measurements, since their readings differ highly at SSCs above  2 g/L (Fig. 2a and d) and the CUS52D inexplicably plateaus after 5g/L. No clear relationship between NTU and SSC can be observed below  2 g/L (Fig. 2b–c and e–f) for all three sensors, even if all sensors are from the same manufacturer and have been calibrated from 0 to 4000 NTU. This is important because many alpine river applications have such low SSCs. Finally, none of the E &H sensors are able to give sensible results for the entire 0–16 g/L range. Further details on the E &H performance are in Supplementary Fig. S11.

### Sensor uncertainty

**Figure 3:**
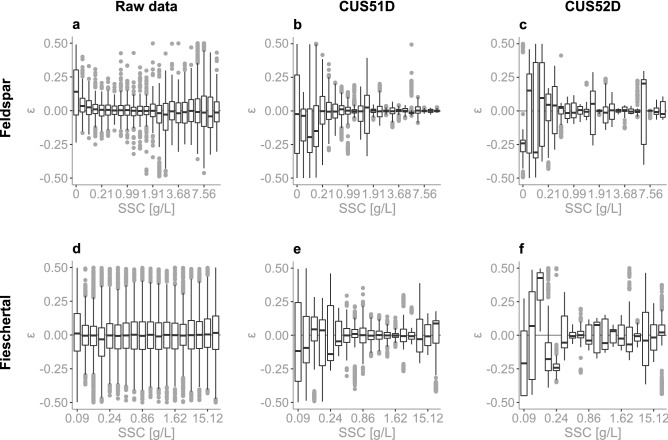
Measurement uncertainty vs SSC in Feldspar (TOP) and Fieschertal (BOTTOM). Uncertainty is defined by $(NTU_i-\overline{NTU})/\overline{NTU}$. Raw data (**a** and **d**) represents the uncertainty at each SSC level for all eight of our open-source sensors. CUS51D (**b** and **e**) represents the uncertainty at each SSC level for both CUS51D sensors. CUS52D (**c** and **f**) represents the uncertainty at each SSC level for the one CUS52D sensor. The boxes represent the interquartile range (IQR), the black line within the boxplot is the median, the bars extend to ± 1.5*IQR, and the grey points are outliers.

An important characteristics of a sensor is its measurement uncertainty, i.e. fluctuations in measurements in time for a given sediment concentration. Figure 3 reports this uncertainty for each SSC level in both Feldspar and Fieschertal sediments, where the measurement uncertainty is defined by:

1 $$\varepsilon = \frac{{NTU_{i} - \overline{{NTU}} }}{{\overline{{NTU}} }}$$

where $NTU_i$ is a single NTU measurement and $\overline{NTU}$ is the mean of all $NTU_i$ measurements (at a specific SSC level). All of the values that were recorded over the length of the experiment were used to plot this figure. On average, for every SSC level the open-source sensors made 91 and 425 measurements in Feldspar and Fieschertal sediment, respectively (sampling rate of ~1 Hz but the sensors were put to sleep periodically during the Feldspar experiments). Whereas for every SSC level the E &H sensors made on average 1421 and 1013 measurements in Feldspar and Fieschertal sediment, respectively (sampling frequency of 1 Hz). Raw data in Fig. 3a and d represents the uncertainty at each SSC level for all eight of our open-source sensors combined. CUS51D (Fig. 3b and e) represents the uncertainty at each SSC level for both CUS51D sensors. CUS52D (Fig. 3c and f) represents the uncertainty at each SSC level for the one CUS52D sensor. The mean and median are identical if the distribution of the uncertainties are symmetric, which is the case for our sensors for most of the concentration range. However, the mean and median disagree for very low and high sediment concentrations, where the uncertainty distribution is non-symmetric.

The uncertainty reported by the open-source sensors increases as SSC increases in Feldspar powder and we see that there are clear sediment-type dependent differences for both our and the commercial sensors. However, our open-sensor results are much more consistent (both sediment types have fluctuations within ±10% (Fig. 3a and d) of the mean) than the E &H sensors that have extremely large fluctuations.

**Figure 4:**
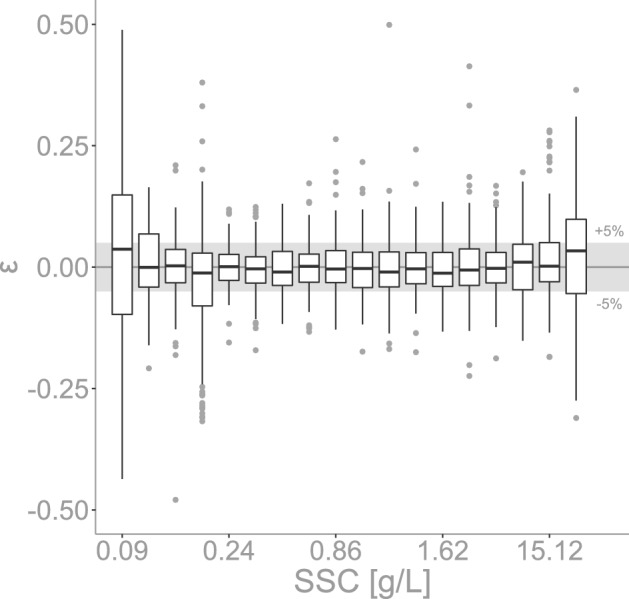
Processed measurement uncertainty vs SSC in Fieschertal sediment. Uncertainty is defined by $(NTU_i-\overline{NTU})/\overline{NTU}$. The plot represent the uncertainty at each SSC level for all eight sensors combined. To obtain this processed data, the mean was taken over bins of 20 measurements (of the raw data from Fig. 3d). The boxes represent the interquartile range (IQR), the black line within the boxplot is the median, the bars extend to ± 1.5*IQR, and the grey points are outliers.

In practical applications we envision that the sensors will collect raw data at a chosen frequency, and this data will be internally processed by taking the mean over a sampling interval, to be stored in the logger (SD card). Figure 4 shows the uncertainty from the processed data for Fieschertal sediment. The processed data in this case was obtained by taking the mean over bins of 20 measurements from the raw data, which would be a typical application in field settings with sampling interval 20s (measurement at 1Hz). This way, we can expect most of our data (IQR) to land between ± 5% of the mean, showing the importance of averaging to reduce variability as opposed to trusting single measurements.

Note in Fig. 4 that the measurement uncertainty is very stable over a large range of SSCs. This consistency is a desired property of the sensors as we expect large fluctuations in SSCs in real river applications. For example, the mean SSC observed across 13 Swiss suspended sediment monitoring stations is 0.11g/L. However, we will be using these sensors in Alpine rivers that are transporting fine sediment and not those that are always have clear water. Therefore, the mean SSC of 11 rivers, excluding the two rivers with the lowest SSC, is 0.129 g/L. The measurement that is exceeded on average 5% of the time is about 0.4 g/L and all of the maximum events fall below 16 g/L except for three events (at 23 g/L, 55 g/L and 71.4 g/L). For more details please see Supplementary Fig. S12. This is well within our tested range and gives us confidence that we can capture well both low and high sediment transport conditions.

### Calibration of new sensor to SSC

NTU as a unit of measure of turbidity is not intuitive for monitoring SSC, as this unit is not universally comparable between watersheds and sensors; you cannot assume that 1000 NTU in two different rivers (or in the same river but using two different sensors) means that there is the same SSC [g/L] present. This is because turbidity does not only depend on SSC, but also on the Particle Size Distribution (PSD), shape, and particle material properties such as colour (reflectivity), density, refractive index, and surface roughness^47–49^. This holds in general and also affects our sensors, however our version A and B sensors overcome this issue of repeatability at least within the same sediment type (Fig. 2).

Observing our open-source sensor performance in Feldspar (Fig. 2a–c), we see that the relationship between NTU and SSC is linear in the sub 2 g/L range and becomes non-linear with increasing SSC. This is in line with what was observed by Holliday^50^ and several others^51,52^. Kelley^43^ tried to use a linear model to calibrate NTU and SSC but had to split their model into several ranges of NTU. Holliday^50^ found through experiments that the relationship between turbidity and SSC follows:

2 $$\begin{aligned} SSC = a(NTU)^b \end{aligned}$$

where *a* and *b* are regression-estimated coefficients and *b* is approximately equal to one for all particles. But in general, for every watershed and sediment type, it is likely that both *a* and *b* will be different. For example, Costa^13^ found $a=0.56$ and $b=1.25$ in their investigated alpine catchment whereas Felix^53^ found $a=0.59$ and $b=1$ in their HPP storage tunnel. In our case, at larger SSCs the relationship diverges from linearity (Fig. 2). Therefore, when working with these sensors, its important to realize that a doubling in SSC can mean a doubling in NTU at lower NTUs and a quadrupling at higher NTUs (for example, 1–2 g/L can give 100–200 NTU but 10–20 g/L can give 1000–4000 NTU).

It is known that the NTU-SSC relationship will depend on sediment type. According to Sadar^41^ and Tran^54^, particles with sizes much smaller than the wavelength of the incident light will scatter light with roughly equal intensity in all directions. Particles larger than the wavelength of the incident light will create a spectral pattern that results in greater light scattering in the forward direction than in the other directions. Therefore, the reported differences in NTUs in Feldpar and Fieschertal sediment (Fig. 2) are likely due to the shape and size of the two sediment types. This shows the importance of calibrating the sensors with sediment samples collected at the river where the sensors would be installed.

**Figure 5:**
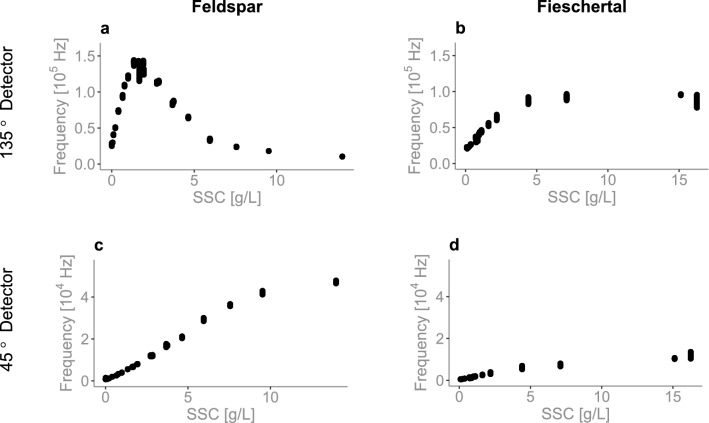
Measured light intensity vs. SSC comparison. Light intensity (measured by the detectors in Hz) shown for the two detectors (135$^o$ and 45$^o$) in Sensor 5 for Feldspar sediment (**a** and **c**) and Fieschertal sediment (**b** and **d**).

Our eight open-source sensors were in a first step calibrated using several dilutions of 4000 NTU Formazin and deionized water: 0, 3, 6, 10, 40, 70, 100, 400, 700, 1000, 2000, and 4000 NTU, as is commonly done in turbidity sensors. However, the sensors calibrated in this way (see Supplementary Methods—First Prototype section), failed to produce data measured in Feldspar and Fieschertal sediment. This is because Formazin scatters light evenly in all directions, which is not true in natural sediment where the directionality of scattered light is highly sensitive to particle grain size. This is demonstrated in Fig. 5, where a peak in light detected by the 135$^o$ detector in Feldspar powder (Fig. 5a) can be observed, whereas in Fieschertal powder (Fig. 5b) there is no peak and the amount of light detected plateaus around 5 g/L. The 45$^o$ detector (Fig. 5c–d) shows a linear increase in the amount of light received in both sediments but the quantity of light received by the detector in Feldspar (Fig. 5c) is much higher than in Fieschertal sediment (Fig. 5d). Similar results were found previously^52^ and similar figures for Formazin are presented in Supplementary Fig. S6.

For this reason, in a second step we developed the Formazin calibration model for the mixing tank experiments (for the results shown in Figs. 2, 3, 4) using only the 90$^o$ (versions A and C) and 45$^o$ detectors (version B) in four distinct NTU ranges (0–10, 10–100, 100–1000, 1000–4000 NTU). The model using only one detector in each sensor (used in Figs. 2, 3, 4) is of the form:

3 $$\begin{aligned} NTU = \alpha + (\beta \times d) + (\gamma \times d^2) \end{aligned}$$

where $\alpha$ is the y-intercept, $\beta$ is the first order coefficient associated with the measured light intensity *d* from the $45^o$ or $90^o$ detector ($90^o$ in versions A and C, $45^o$ in version B) and $\gamma$ is the second order coefficient from the same detector. The resulting models for Sensor 6 for the four different NTU ranges (0–10, 10–100, 100–1000, and 1000–4000 NTU) are shown in the Supplementary Fig. S13 and their coefficients are in Supplementary Table S5.

Finally, we challenge the need to calibrate the sensors to NTU in order to predict SSC from a separate NTU-SSC relation, which compounds estimation errors. Our sensors are meant for use in a range of different rivers with various grain sizes and shapes (therefore, constantly changing the direction of scattered light). We envision that one can directly calibrate SSC observations to the reflected light intensity observed by the combined detectors. This can be done in laboratory calibration experiments or in on-site river applications by measuring suspended sediment concentration in a few samples that cover a range of SSCs.

**Figure 6:**
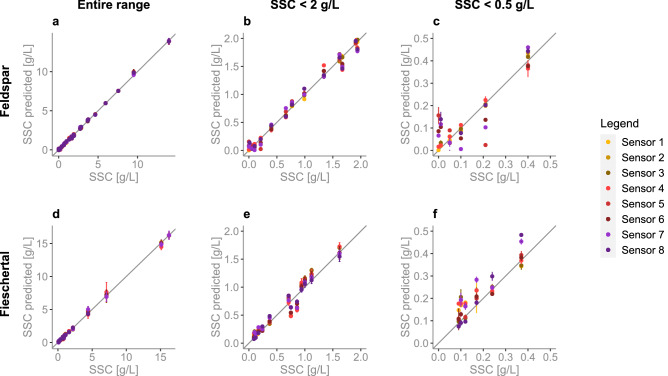
SSC measured vs. mean predicted in three different ranges of Feldspar (**a**–**c**) and Fieschertal (**d**–**f**) sediments as predicted from a fourth order multiple linear regression SSC model created for each of our open-source turbidity sensors (all of them $R^2 > 0.984$). Version A sensors are in yellow gradient (Sensor 1 in bright yellow, Sensor 2 in mid-yellow, Sensor 3 in dark yellow). Version B sensors are in red gradient (Sensor 4 in bright red, Sensor 5 in mid-red, Sensor 6 in dark-red). Version C sensors are in purple gradient (Sensor 7 in bright purple, Sensor 8 in dark purple). The error bars are ± one standard deviation.

In this approach we calibrated the light intensity measured by the detectors for each open-source sensor directly to SSC, separately for the Feldspar (Fig. 6a–c) and Fieschertal (Fig. 6d–f) sediment. The model used is of the form:

4 $$\begin{aligned} SSC = \alpha + (\beta _1 \times d_{1}) + (\beta _2 \times d_{2}) + (\gamma _1 \times d_{1}^2) + (\gamma _2 \times d_{2}^2) + (\delta _1 \times d_{1}^3) + (\delta _2 \times d_{2}^3) + (\eta _1 \times d_{1}^4) + (\eta _2 \times d_{2}^4) \end{aligned}$$

where $\alpha$ is the y-intercept, $d_1$ is the light intensity measured by the 45$^o$ or 90$^o$ detector (90$^o$ in versions A and C, 45$^o$ in version B), $d_{2}$ is the light intensity measured by the 135$^o$ detector (in versions A-B-C), $\beta _{1,2}$-$\eta _{1,2}$ are the 1st-4th order coefficients associated with $d_1$ and $d_2$. Figure 6 shows SSC mean predictions against observations for all versions of the sensor. The fit is excellent across the entire range of SSC with $R^2>0.98$, with the main benefit due to the multiple linear regression using both detectors. Analysing the versions separately, all sensors are now able to predict well in the entire SSC range down to 0.4 g/L in Feldspar (Fig. 6c, with version A performing well down to 0 g/L) and down to 0.25 g/L in Fieschertal (Fig. 6f, with versions A and B performing well down 0.12 g/L and 0.17 g/L, respectively). Here the 3D printed sensors (version C) do not perform as well. An improvement in the 0–0.5g/L range can probably be done by splitting the model and having two separate linear calibrations. The advantage of the open-source sensors is that the user does not need to use the 4th order model as we have done, and is free to chose their own model.

It is important to note that avoiding the Formazin calibration step has additional benefits. By calibrating from the detector output directly to SSC, we are able to save up to a week’s worth of lab work and we are able to avoid exposure to Formazin, a known carcinogen. Collecting gravimetric samples and calibrating directly to SSC might seem like a lot of work, but this needs to anyway be done when installing turbidity sensors in a river network to collect SSC data. Additional problems with Formazin calibration can be found in Supplementary Discussion–Problems with calibrating NTU with Formazin.

**Figure 7:**
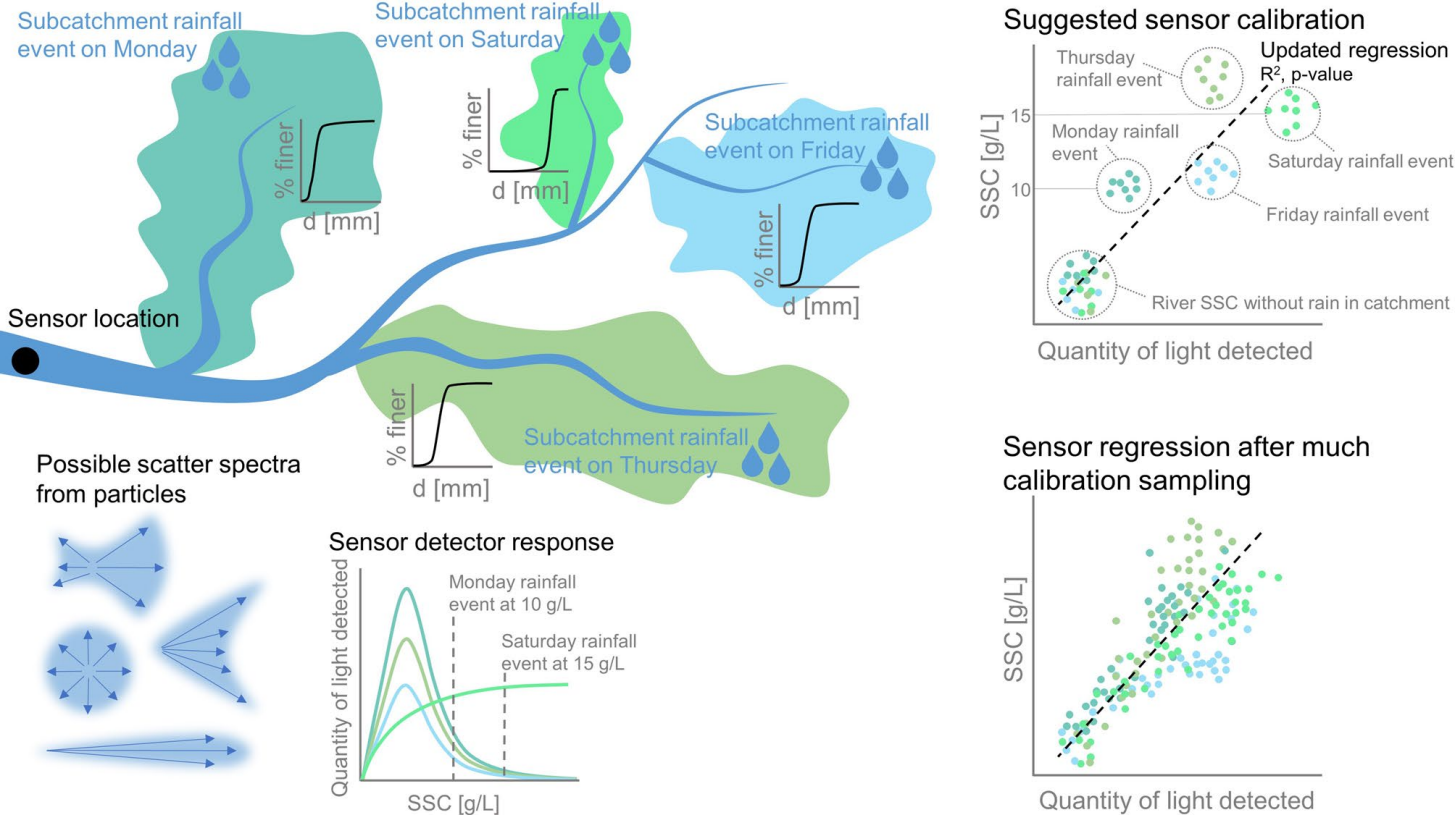
Conceptual scheme of a direct calibration of SSC to the light detected by a sensor in a river with several subcatchments that all have different sediment types and grain size distributions and therefore, emit different scatter spectra. In this example, every subcatchment experiences an isolated rainfall event on a different day. The sensor detectors at the downstream end of the river have a different light intensity response depending on the sediment type. After sampling many different events, a single calibration curve representative of the different subcatchment sediment sources and their activation frequencies can be obtained.

A conceptual example of how direct calibration of the open-source sensor to SSC would work in a real case and how this calibration gives a more representative SSC reading is shown in Fig. 7. From Monday to Saturday there are isolated rainfall events which occur only in certain subcatchments, as depicted in Fig. 7. Each subcatchment has a different grain size distribution and different grain properties, which in turn scatter light differently (creating different scatter spectra). A sensor is located near the outlet of a catchment. The sensor works by measuring the quantity of scattered light (at a specific angle) and it is consistently measuring this value only. A rainfall event on Monday in a subcatchment with very fine particles gives rise to an SSC of 10g/L at the sensor location (gravimetrically determined SSC from a bottle sample). However, these fine particles do not scatter light strongly in the direction of the sensor detector. This causes the calibration points to sit above our calibration curve (low light detected - similar to Feldspar). Whereas, a rainfall event on Saturday in a subcatchment with very coarse particles, giving rise to an SSC of 15g/L, scatters light strongly in the direction of the sensor detector (even more than is expected at this SSC). This causes the calibration points to sit below our calibration curve (higher amount of light detected - similar to Fieschertal).

The combination of response curves (Fig. 5) from multiple detectors can produce good reflectance curves for a range of sediment types (sources) and concentrations (Fig. 6). The mixing of these response curves over many events leads to a robust calibration curve for the entire catchment. However, the measurements of individual flood events will have errors when using a sensor at the outlet of the catchment. As our sensors can be used as cheaper alternatives, this limitation is overcome by placing several of these sensors within subcatchments in order to narrow the uncertainty in SSC. By calibrating the affordable sensors to the SSC of each individual subcatchment, our sensor response would be unique to the sediment type of the subcatchment. In this way, sediment sources with possibly different sediment properties may be identified.

## Outlook

In the future, we would like to incorporate a temperature and pressure sensor into our sensors to also monitor the stage and temperature of the river. A wiper is absolutely essential in the sensor design for long-term monitoring. We will be sure to include it in our next prototype. The Hz-SSC calibration curve, along with the number of sediment-water samples necessary to create this curve, should also be investigated further. It is clear that over a longer timescale that the catchment properties will change, causing a change in the Hz-SSC calibration curve (which is also the case for traditional NTU-SSC turbidity sensors). Therefore, our sensors are not intended to replace long-term river monitoring, but rather provide short-term identification related to changes in water quality, and finding sediment sources and their activation.

Our hope is that the affordable, open-source SSC sensor brings accessibility to global river research. Blog posts with fabrication instructions can be found on our website^55^. With a fully transparent design, students, researchers, and organizations are able to build, install, use, and repair the instruments themselves, ultimately eliminating waste and making the data from our world’s rivers, lakes, and oceans available to all.

## Methods

**Figure 8:**
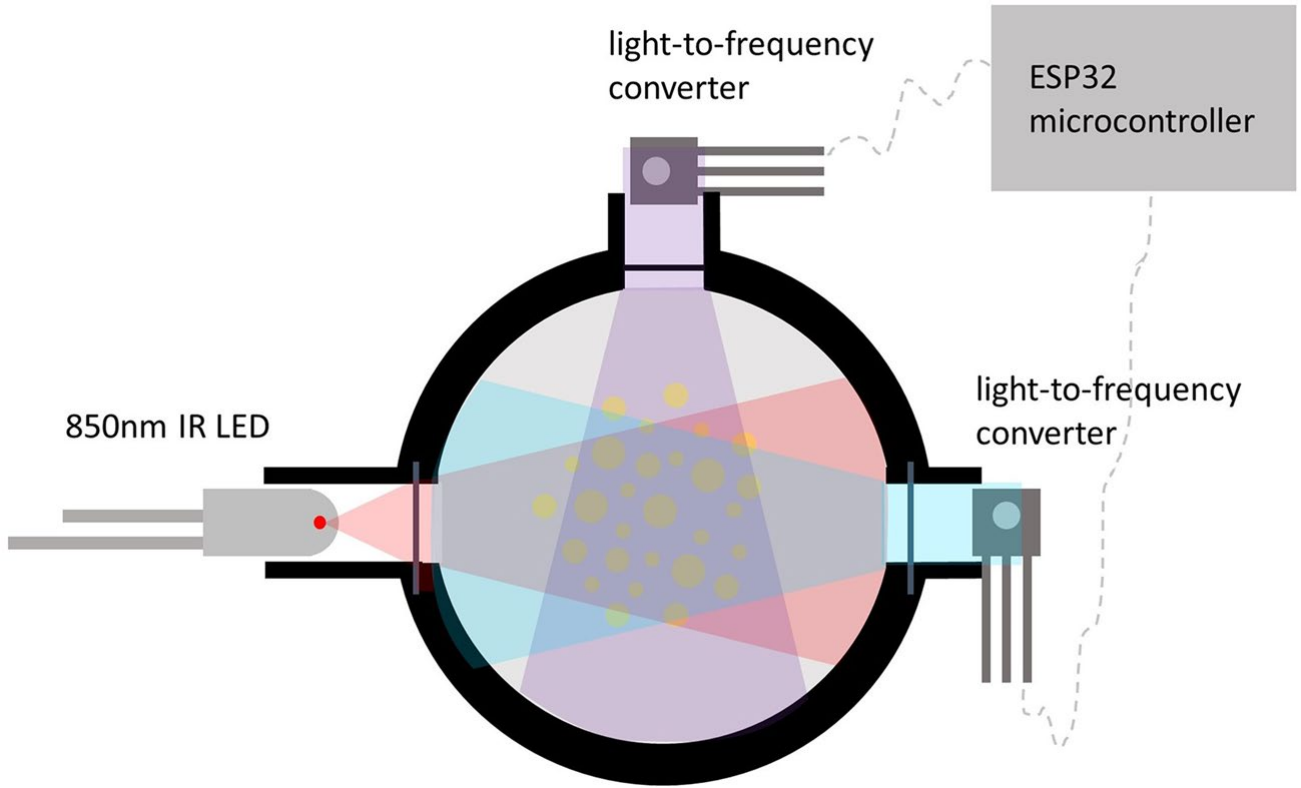
Sensing principle behind the open-source turbidity sensor.

The basic design of our turbidity sensor was created with an infrared (IR) LED and two light detectors at different angles relative to the LED. The design principle can be seen in Fig. 8. An LED at 850nm was selected (TSHG6200 by Vishay Semiconductor Opto Division) because water as a medium does not reflect this wavelength. The detectors chosen are the TSL237S-LF by AMS and they were chosen for two reasons: (1) they convert light to a digital output frequency and so variations in voltage or current driven by temperature fluctuations in the field environment, that affect an analog detector, are eliminated; (2) this sensor responds to light in the 320nm to 1050nm range and has a peak responsivity at 700nm. An ESP32 was chosen as the microcontroller for this sensor instead of an Arduino Uno (a common microcontroller chosen for hobbyists) because of its superior number of timers. Since the detectors output a digital pulse with varying frequency proportional to the light intensity, the chosen microcontroller should have at least two timers to compute frequency, one to count pulses and one to count the elapsed time, for each detector. Therefore, the Arduino can only be used to convert the digital output of one detector and the ESP32 is needed because it has the required four timers to resolve the digital signal of two detectors.

With the above design in mind, we built several versions of our first turbidity sensor prototype and tested it with Formazin, a popular turbidity sensor calibration liquid. Details about focusing lenses, ambient stray light elimination, detector placement, and calibration model can be found in the Supplementary Methods–First prototype section.

In order to test the turbidity sensors in a real-world setting, we designed a mixing tank experiment where our sensors can be compared to commercially-available sensors. In order to submerge our sensors into a mixing tank and take many measurements over several hours, a second prototype of the turbidity sensor was created.

### Second prototype construction

From the Supplementary Methods—First Prototype, it was found that focusing lenses were necessary for the design of the sensor, whereas long-pass filters (eliminating stray ambient light) were unnecessary since the same effect is achieved using the off-on differencing measurement method^56^. Additionally, for our chosen LED and detector parts, it is important to avoid the 180$^o$ detector placement since the detector becomes saturated by the incoming light (see Supplementary Methods—First Prototype for more details).

With this in mind, the second prototype was based off of Sensors 3 and 4 found in Supplementary Methods–First Prototype section because we wanted to test the detector orientations further. It has three different versions (A, B, and C) and 2-3 replicates of each version (to see the variation from construction) and all of the sensors and replicates are summarized in Supplementary Table S4. Version A has detectors at $90^o$ and $135^o$ relative to the LED and version B has detectors at $45^o$ and $135^o$. Unlike the first prototypes created in the Supplementary Methods–First Prototype section that had a 3D-printed sensing head, these first six sensors have sensor heads that were made by machining a solid piece of black PVC. However, with the hope to keep this project accessible to everyone, we also created two additional replicates (version C) but with a 3D-printed sensing head from PLA. All eight sensors details are listed in Supplementary Table S4.

**Figure 9:**
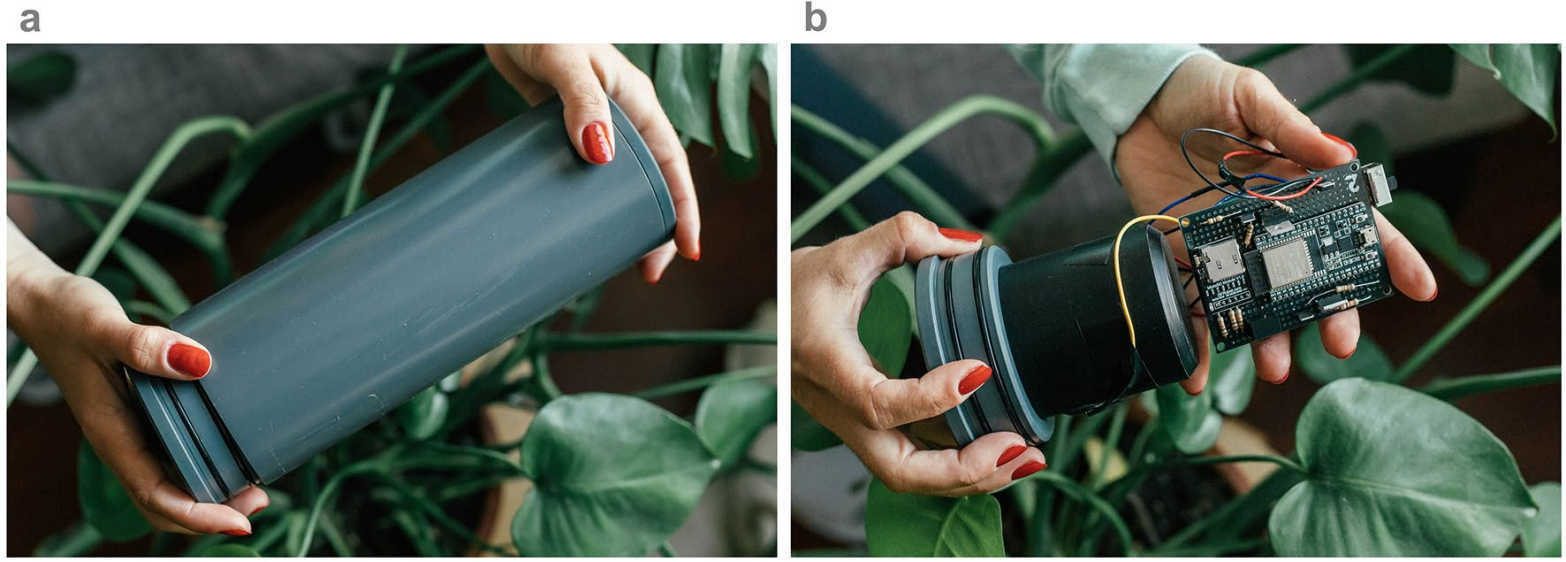
Open-source turbidity sensor—second prototype. (**a**) Closed sensor with external PVC housing. (**b**) Internal electronics of the sensor.

All eight sensors were housed in a simple waterproof housing made of standard PVC from the hardware store. The waterproof housing can be seen in Fig. 9a. Figure 9b shows the internal sensing head (black PVC) and the electronics of the sensor. We also see two O-rings on the left-hand side of both images. These, along with some vacuum grease, were used to keep the sensor waterproof. All of the electronics of the sensor were housed on a prototyping soldering board. The electronic schematic is found in Supplementary Fig. S14 and the CAD for the PVC sensing head is available on this project’s repository^57^.

### Commercial sensors

We tested three commercial turbidity sensors against our open-source sensors. These sensors were two CUS51Ds and one CUS52D, all from Endress+Hauser (E &H). The CUS51Ds are rated for a measurement range of 0 to 4000 FNU and are used for turbidity and suspended solids measurement at all stages of the wastewater treatment process and at primary water treatment applications with medium to high turbidity^58^. The CUS52D is also rated for a measurement range of 0–4000 FNU, but unlike the CUS51D, the CUS52D can be used at every stage of the water treatment process, even at the lowest turbidity^59^. The E &H sensors additionally needed to be hooked up to a data acquisition system (Liquiline CM442), which enabled the sensors to continuously measure while connected to a power source.

### Cost

All of the parts purchased to make one of our open-source sensors are shown in Supplementary Table S6. The total cost of one sensor is 61.37 CHF (Swiss Francs). It is worth noting that the cost of our sensor doesn’t include calibration labor costs and we are not sure if the E &H sensors must undergo additional calibration and/or servicing prior to every use. Machined PVC is more expensive and harder to produce than simply 3D printing the sensing head. Therefore, before deciding to 3D print or purchase and machine PVC, the purpose of the sensor should be evaluated. If replicates of the sensor need to be made in order to create a distributed network of sensors within a watershed, then perhaps purchasing and machining PVC is worth the extra effort in order to have comparative results along the watershed. However, if only one sensor needs to be created and installed at the outlet of a water system in order to obtain a basin-integrated picture of the suspended sediments, then a 3D printed sensor is probably enough for this purpose and the extra time and cost would not contribute much to the final output. The 3D printed version can also be used to monitor trends in a distributed network instead of absolute values. Additionally, if the sensor is used for a school project then a 3D-printed sensor should suffice.

### Mixing tank setup

**Figure 10:**
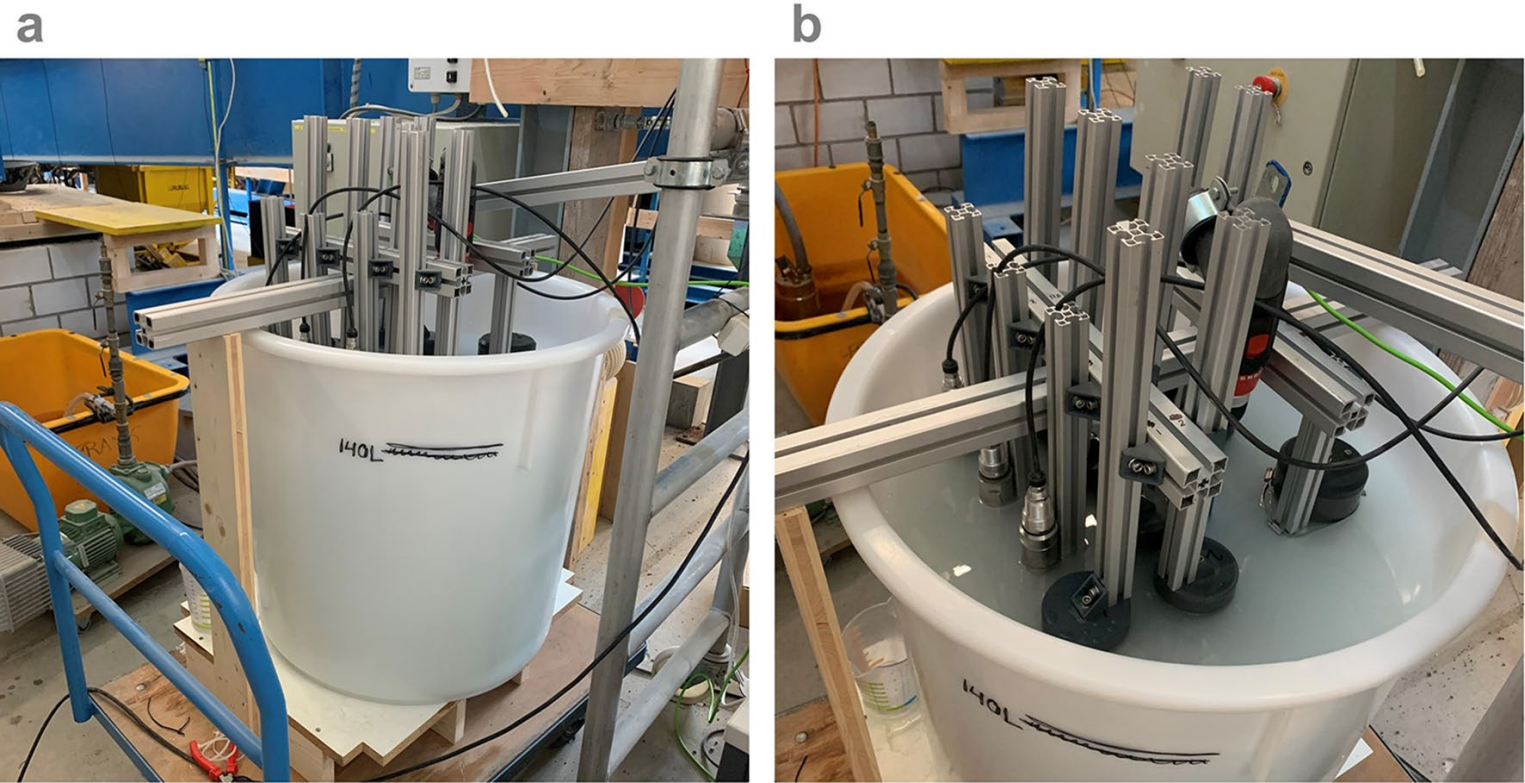
Mixing tank setup: (**a**) cylindrical 200L tank, (**b**) arrangement of 11 sensors (8 of ours and 3 commercial) within the tank and the mixing drill in the middle.

Figure 10a shows the mixing tank setup with a 200L cylindrical tank and a line marking 140L of water when the tank is full with all 11 tested sensors. In the background, the green pump used to empty the tanks can be seen. Figure 10b shows the placement of the sensors around the tank along with the water level. In the center of the tank is the mixer, which is a drill with a paint mixing attachment. The mixer was used to suspend the sediment in the tank.

### Sediment type

There were two different sediment types used in these experiments: Feldspar and sediment taken from the Fieschertal canal, both of which were used previously by Felix^44^.

Feldspar powder (Na-plagioclase) was chosen because of its abundance in the earth’s crust. It was purchased from a manufacturer that sells Feldspar milled for pottery (Feldspat NA LF 90). The Fieschertal sediments were collected from the deposits in the tailwater channels at the Fieschertal hydropower plant (HPP) in the Canton of Valais, Switzerland. This sediment was chosen because we are planning on using our open-source sensors to investigate sediment sources on the Rhone river such as the Fiescher glacier. The Fiescher HPP is located at the mouth of the Fiescher glacier, on the mountain stream Wysswasser which is a tributary of the upper Rhone. However, it should be noted that the finer sediments do not settle in the tailwater channel, therefore the Fiescher sediments are coarser than would be expected.

The particle size distribution (PSD) and the solid densities, $\rho _s$, of both powders were measured previously by Felix^44^. The PSD was measured from suspended samples with adequate dilution using a stationary laser diffractometer (LA-950 manufactured by Horiba). It was found that the Fieschertal sediments contained almost 80% by mass of fine sand ($d_{50}=90 \mu m$) whereas the Feldspar was mainly in the range of silt ($d_{50}=30 \mu m$)^44^. The solid densities were measured using a helium expansion pycnometer and are $2.65g/cm^3$ and 2.70 $g/cm^3$ for the Feldspar and Fieschertal sediment, respectively^44^.

### Experimental procedure

The experimental procedure was as follows. First, all of the sensors would be turned on and the clean tank would be filled with tap water. The E &H sensors take a measurement every second for the duration of the experiments. For the first experiment with the Feldspar, the open-source sensors were taking 3 measurements for 3 seconds every minute and sleeping between readings. For the next experiment with the Fieschertal sediment, the open-source sensors were taking a measurement every second (as the E &H sensors) without sleeping between readings.

With clear water in the tank, the mixing drill and measurements were started. Every 30 min afterwards, the water temperature of the tank was taken along with a water sample of around 300mL. Afterwards, a pre-defined amount of Feldspar sediment mixed with 300mL of tap water was added to the tank in order to increase its SSC in a step-wise manner. This experiment was repeated again for the Fieschertal sediment except since the open-sourced sensors were continuously measuring like the E &H devices, we were able to increase the SSC of the tank every 15 min instead of every 30 min. Every time we increased the SSC of the tank, we also wiped all of the bubbles off of the 11 sensors.

Afterwards, the water-sediment samples were taken to the lab and evaporated in a ventilated oven and the SSC was computed by weighing the dry sediment. Some tap water samples without sediment were also evaporated so we could determine the dissolved mineral concentration and subtract this from our calculated SSC.

## Supplementary Information

Supplementary Information.

## Data Availability

The datasets generated during and/or analysed during the current study are available in the Zenodo repository, 10.5281/zenodo.5789513.
