# Supplementary material for: Open-source, low-cost, in-situ turbidity sensor for river network monitoring
Source: Sci Rep. 2022 Jun 20;12:10341. doi: 10.1038/s41598-022-14228-4 (PMC9209475; doi:10.1038/s41598-022-14228-4)
Supplement: Supplementary file 1 — Supplementary Information. [file 41598_2022_14228_MOESM1_ESM.pdf]

Supplementary materials

Supplementary Methods - First Prototype

A first prototype of the sensor was created. The sensor's head, which houses the LED and detectors, was 3D printed and its drawing is shown in Fig. S1 RIGHT. Black filament was chosen to prevent internal reflections within the sensor head. The 3D printed sensor head was attached to a grey PVC tube (Fig. S1 LEFT) in order to minimize stray ambient light and to hold a larger sample volume of calibration liquid. Attached to the sensor head and grey PVC tube is a prototyping breadboard which held the electronic components; all three are shown in Fig. S1. All of the CAD files can be found at this project's repository[1].

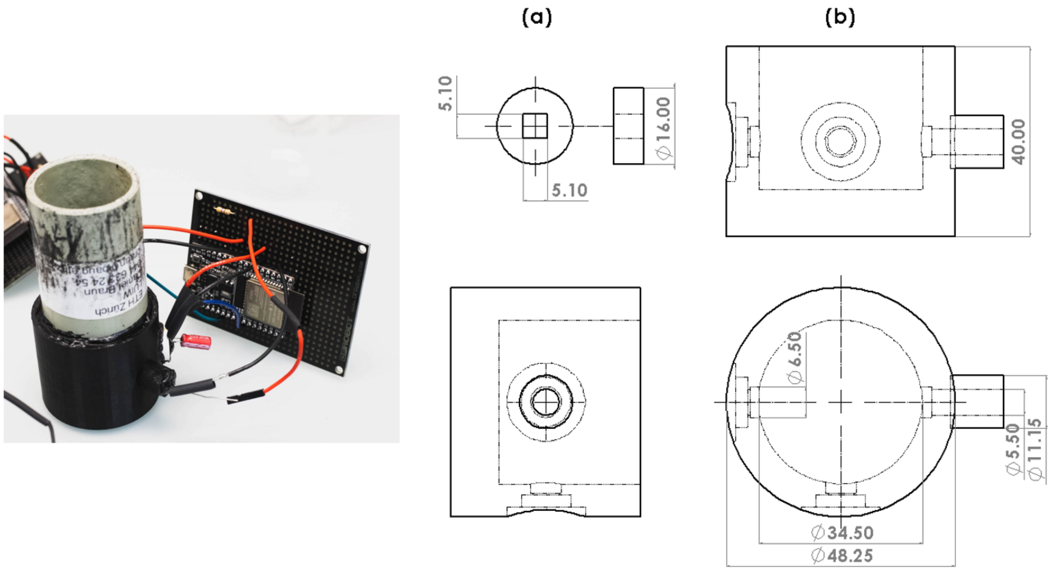

Figure S 1. LEFT: First prototype of the turbidity sensor, RIGHT: drawing of the 3D printed sensing head.

Four different versions of this turbidity sensor were created and are summarized in Table S1. The first sensor (Sensor 1) is one with detectors at 90° and 180° and flat glass separating/protecting the LED and detectors from the liquid sample. The second sensor (Sensor 2) has detectors again at 90° and 180° but with plano-convex focusing lenses instead of flat glass. These selected focusing lenses are made of H-K1 glass with a 6mm diameter and an 8.873mm focal length. The third sensor (Sensor 3) has detectors at 90° and 135° and again plano-convex lenses. It was also built in a manner to insert/remove long-pass filters. The long-pass filters selected pass everything longer than 750nm, therefore all visible light should not be able to reach the detectors whereas the 850nm LED signal should be able to reach the detectors. Finally, the fourth sensor (Sensor 4) has detectors at 45° and 135° and the same plano-convex lenses.

Table S 1. List of sensor versions.

Sensor version	Detector Orientations	Type of lens
1	90°, 180°	no lens
2	90°, 180°	lens
3	90°, 135°	lens + insertable long-pass filters
4	45°, 135°	lens

In order to take a measurement with these sensors, a sample is poured into the grey PVC + 3D printed sensing head (Figure S1 LEFT). Since the detectors output a digital frequency, a pulse-counting program was written and uploaded onto the ESP32 to count the pulses and compute the frequency from both detectors simultaneously. This program was based off of David Antliff's *frequency_count.h* library and the full code can be found on this project's repository[1]. The principle behind the pulse-counting program is that it uses one timer to count the number of digital pulses while the second timer simultanuously counts the amount of time elapsed. This then gives one frequency datapoint in hertz. For all of our measurements, we consider one frequency datapoint to be an average of 10 frequency measurements (number of pulses divided by the elapsed time) taken

in the span of a few miliseconds.

Formazin calibration

In order to select the version of the sensor that works best for turbidity measurements, three different experiments were run and are summarized in Table S2. The first experiment, illustrated in Figure S2, was to see the effect of including the focusing lens in front of the LED and detectors (using Sensors 1 & 2). The second experiment, illustrated in Figure S3, was to determine which detector orientations relative to the LED gave the best results in the full 0-4000 NTU range (using Sensors 2, 3 & 4). Three detector orientations relative to the LED were tested: 90° and 180°, 90° and 135°, and 45° and 135°. Figure S4 illustrates the third experiment, which was used to determine the effect of ambient light leakage and whether the long-pass filters could minimize the ambient light interference (using Sensor 3 with & without the long-pass filters).

Table S 2. List of experiments.

Experiment	Sensor name	Lens [Y/N]	Detector orientations	Filters [Y/N]	Ambient light [Y/N]
1	1	N	90°, 180°	N	N
	2	Y	90°, 180°	N	N
	2	Y	90°, 180°	N	N
2	3	Y	90°, 135°	N	N
	4	Y	45°, 135°	N	N
3	3	Y	90°, 135°	N	Y
	3	Y	90°, 135°	Y	Y

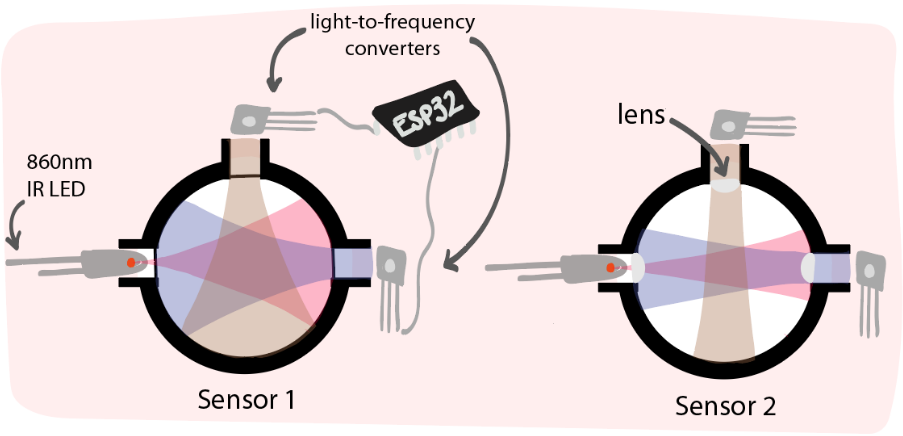

Figure S 2. Lens effect experiment. LEFT: sensor without lenses, RIGHT: sensor with lenses.

Several dilutions of Formazin, a popular calibration liquid for turbidity sensors, were created. The Formazin purchased was rated at 4000NTU and, using deionized water, dilutions were created: 0, 2, 4, 6, 8, 10, 20, 40, 60, 80, 100, 250, 500, 800, 1000, 2000, and 4000 NTU. This standard calibration liquid was used instead of preparing test solutions using another liquid of unknown NTU and a commercial turbidity sensor to obtain the liquid's NTU value (similar to Gillett[2]). This was done because often these sensors do not report an accurate value for NTU[3] and therefore report unreliable NTU units. Each Formazin dilution was then poured into the Sensors 1-4 in order to carry out the experiments listed in Table S2.

To build a sensor which has high dynamic range, common error compensation (due to water, colour, and attenuation), low sensitivity to the bandwidth of the optical source, and high reliability, Postolache[4] found that a light scattering measurement using the LED and detector(s) should be immediately proceeded by a scattering measurement where the LED is turned off. Matos[5] found that the LED off-on measurement is necessary since this type of optical device is influenced by external light. With this in mind, 100 frequency data points were recorded at 1Hz for each detector, first with the IR LED off, then again with the IR LED on.

From the 100 datapoints, the mean and standard deviations were calculated for both when the LED was off and on. Then the mean values of the LED-off frequency was subtracted from the mean value of the LED-on frequency in order to create the final frequency values for each detector. This was repeated three times at each NTU dilution and for every experiment.

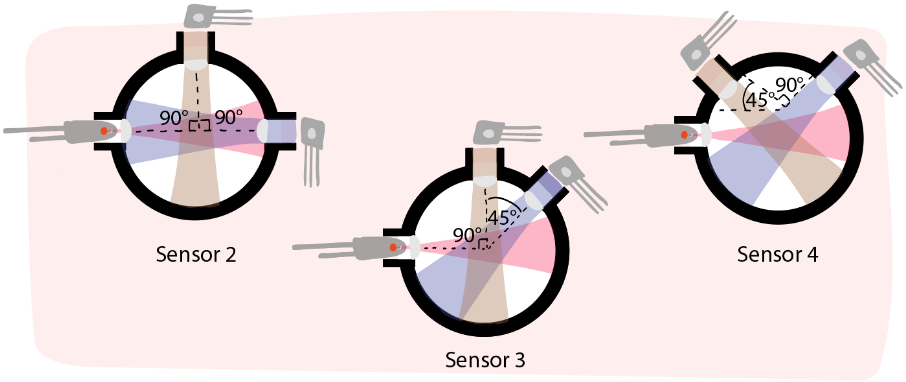

Figure S 3. Detector orientation experiment. LEFT: Sensor 2 with 90° and 180° detectors, MIDDLE: Sensor 3 with 90° and 135° detectors, RIGHT: Sensor 4 with 45° and 135° detectors.

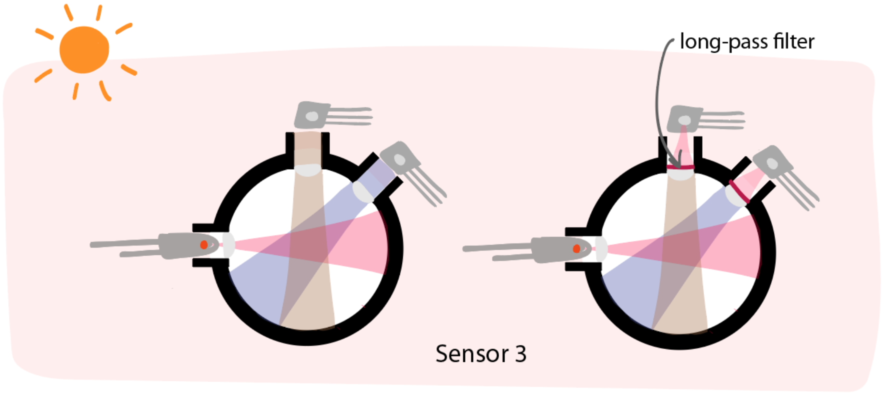

Figure S 4. Ambient light experiment. LEFT: without long-pass filters, RIGHT: with long-pass filters.

Effect of adding focusing lenses

Figure S5 shows the mean and errors (+/- standard deviation) for both the 180° and 90° detectors for Sensor 1 (Fig. S5a) and Sensor 2 (Fig. S5b) for experiment 1.

Figure S5 is reporting on the amount of light (in digital frequency) reaching each detector at any given NTU dilution. Here, we would expect smooth curves[6]. In this figure, we see that there is no clear relationship between frequency in the 180° and 90° detectors and NTU when there are no lenses. When we include lenses (Fig. S5b), both the 180° and 90° detectors begin to have a clear relationship with NTU (similar to the one found in Figure 11 by Sadar[6]), however the 180° detector is cut off at NTUs below 1000 probably because the detector has reached its saturation limit. From these results, it is reasonable to conclude that our sensors require focusing lenses.

Effect of Detector Orientation

Figure S6 shows the mean and errors (+/- standard deviation) for Sensors 2-4 with 180° and 90° detectors (Sensor 2 in Fig. S6 a and d), 135° and 90° detectors (Sensor 3 in Fig. S6 b and e), and 135° and 45° detectors (Sensor 4 in Fig. S6 c and f) in experiment 2.

In this figure, we see that there is a clear relationship between the amount of light reaching the detectors (frequency in hertz) and NTU, similar to those reported by Sadar[6]. Figure S6a again shows that the frequency is cut off below 1000 NTU. Figures S6 b and c report different maxima from the 135° detector, this is probably due to a difference in the sensors' construction and shows the importance of calibration when making these homemade sensors. From these results, it is reasonable to conclude that a sensor with detectors at 45°, 90°, and/or 135° would be suitable for our purposes.

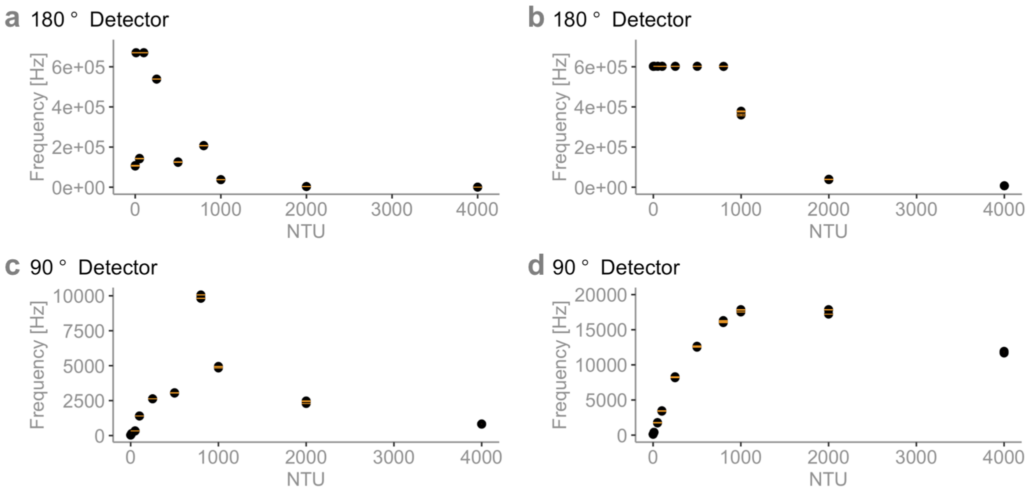

Figure S 5. Quantity of light in hertz [Hz] reaching the detector for each NTU dilution. The mean (black points) and errors (orange bars from +/- standard deviation) for both the 180° and 90° detectors for Sensor 1 without lenses (a and c) and Sensor 2 with lenses (b and d). The mean and error were found by subtracting the mean of when the LED was off to the mean of when the LED was on.

Model creation

Several models were created both for experiments 2 and 3. Figure S7 shows a comparison of four different multivariable models for Sensor 3 (data taken from experiment 2). The models are of the form:

$$NTU = \alpha + (\beta_1 \times d_{90^0}) + (\beta_2 \times d_{135^o}) \quad (1)$$

$$NTU = \alpha + (\beta_1 \times d_{90^0}) + (\beta_2 \times d_{135^o}) + (\gamma_1 \times d_{90^0}^2) + (\gamma_2 \times d_{135^o}^2) \quad (2)$$

$$NTU = \alpha + (\beta_1 \times d_{90^0}) + (\beta_2 \times d_{135^o}) + (\gamma_1 \times d_{90^0}^2) + (\gamma_2 \times d_{135^o}^2) + (\delta_1 \times d_{90^0}^3) + (\delta_2 \times d_{135^o}^3) \quad (3)$$

$$NTU = \alpha + (\beta_1 \times d_{90^0}) + (\beta_2 \times d_{135^o}) + (\gamma_1 \times d_{90^0}^2) + (\gamma_2 \times d_{135^o}^2) + (\delta_1 \times d_{90^0}^3) + (\delta_2 \times d_{135^o}^3) + (\eta_1 \times d_{90^0}^4) + (\eta_2 \times d_{135^o}^4) \quad (4)$$

For the full range of data (Fig. S7a) none of the given models are able to accurately predict NTU, except for the fourth order model which is able to predict down to 250NTU. However, in Fig. S7b, we see that it is unable to predict at NTUs lower than 250. In order to calibrate these sensors in the 0-250NTU range, a separate model needs to be created for this range. The final calibration for this sensor would be a model in the 0-250NTU range and a fourth order model in the 250-4000NTU range. Due to the unnecessary complexity of the fourth order model, we decided instead to fit the data using the simplest multiple linear regression model in four different ranges (0-10NTU, 10-100NTU, 100-1000NTU, 1000-4000NTU), as this is often done by commercial turbidity sensors. The model is of the form:

$$NTU = \alpha + (\beta_1 \times d_{90^0}) + (\beta_2 \times d_{135^o}) \quad (5)$$

where α is the y-intercept, and β_1 and β_2 are the coefficients associated with the first detector (d_{90^0}) and second detector (d_{135^o}), respectively. The new model for Sensor 3 is shown in Fig. S8. The same model was also applied to Sensor 4, which is also shown in Fig. S8 with the model coefficients of both sensors in Table S3.

Effect of ambient light and long-pass filter

Figure S9 shows the mean and errors (+/- standard deviation) for Sensor 3 under ambient light conditions both without and with long-pass filters in front of the detectors, for experiment 3.

From this figure, we see that it is very difficult to notice an improvement in the data from the long-pass filters. Figure S9 shows a slight decrease in frequency when the long-pass filters are applied, as expected.

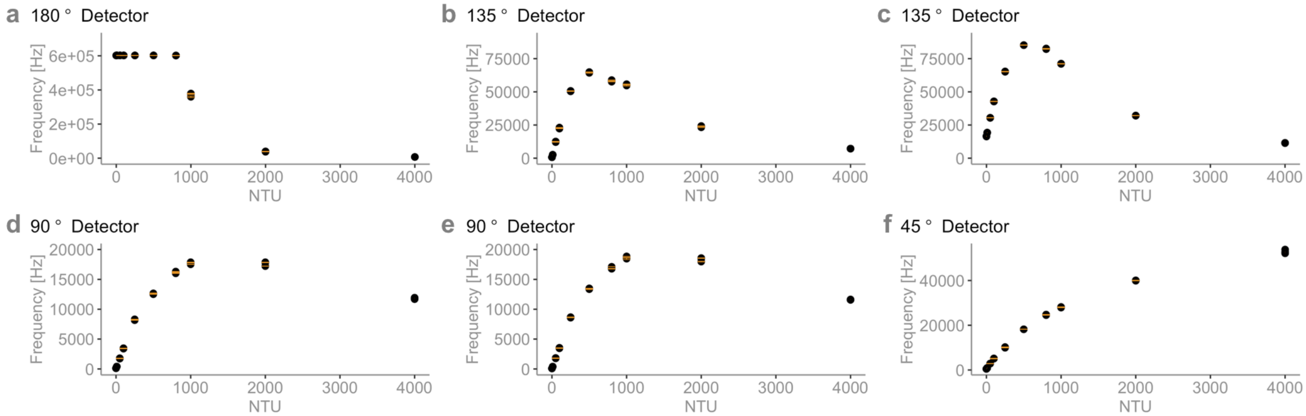

Figure S 6. Quantity of light in hertz [Hz] reaching the detector for each NTU dilution. The mean (black points) and errors (orange bars from +/- standard deviation) for Sensor 2 (a and d: 180° and 90° detectors), Sensor 3 (b and e: 135° and 90° detectors), and Sensor 4 (c and f: 135° and 45° detectors). The mean and error were found by subtracting the mean of when the LED was off to the mean of when the LED was on.

Table S 3. Model Coefficients for Sensors 3 and 4.

Sensor	Range [NTU]	α	β_1	β_2	R^2	p-value
3	0-10	-2.0546316	0.0261069	0.0003605	0.9931	< 2.2e-16
3	10-100	-1.4655810	0.0253945	0.0001387	0.9952	< 2.2e-16
3	100-1000	32.0347677	0.0778393	-0.0090305	0.9981	< 2.2e-16
3	1000-4000	6.773e+03	-2.209e-01	-2.990e-02	0.9978	4.526e-09
4	0-10	-2.222e+00	1.934e-02	-4.128e-04	0.996	< 2.2e-16
4	10-100	-1.150e+01	1.943e-02	-4.556e-05	0.9914	1.453e-14
4	100-1000	49.2023705	0.0436498	-0.0039107	0.9992	< 2.2e-16
4	1000-4000	-7.726e+03	2.129e-01	3.855e-02	0.9928	1.576e-07

Model applied to experiment 3

When applying the same linear model from the previous subsection to the ambient data obtained in experiment 3, we obtain Fig. S10. In this figure we present Sensor 3 exposed to ambient light with (white circles) and without (black triangle) long-pass filters in the 0-10 NTU range (since this range is the most likely to be affected by ambient light). We see that the off-on differencing measurements seem to be enough to eliminate ambient stray light. Additionally, the long-pass filters (white circles) might not have been aligned perfectly when building the sensor and so the predicted NTU is worse (less precise) than the case without filters (black diamond). From this figure and from Fig. S9, we see no reason to opt for the long-pass filters when the off-on measurement is sufficient to eliminate stray light even in the 0-10NTU range. These lenses also add to the cost of the sensor (8 USD extra per sensor, each lens costs 4 USD).

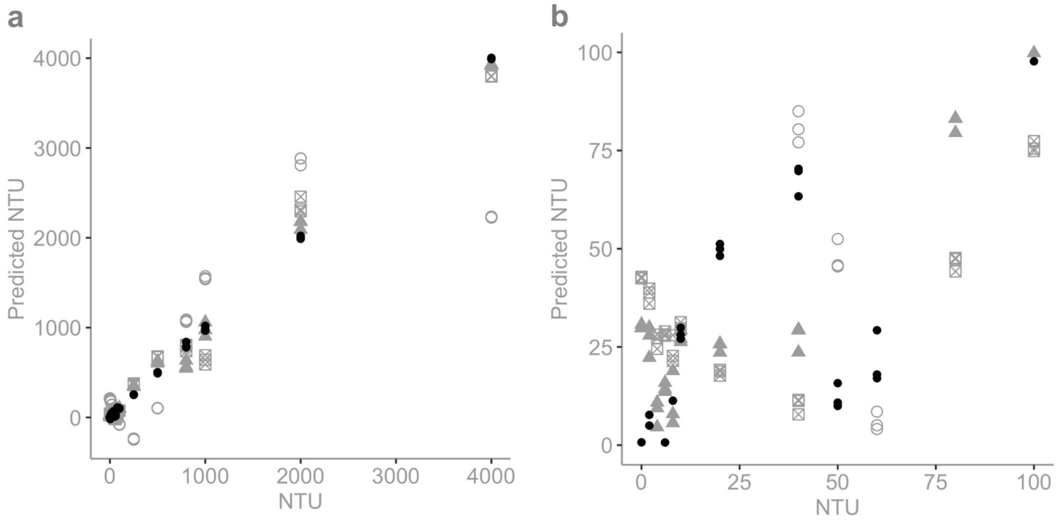

Figure S 7. Comparison of four different multivariable models for Sensor 3: a) 0 to 4000 NTU and b) 0 to 100 NTU. The four different models are on the order of n=1 (equation 1 - grey, hollow circle), n=2 (equation 2 - grey, square with cross), n=3 (equation 3 - grey, solid diamond), and n=4 (equation 4 - black, solid circle).

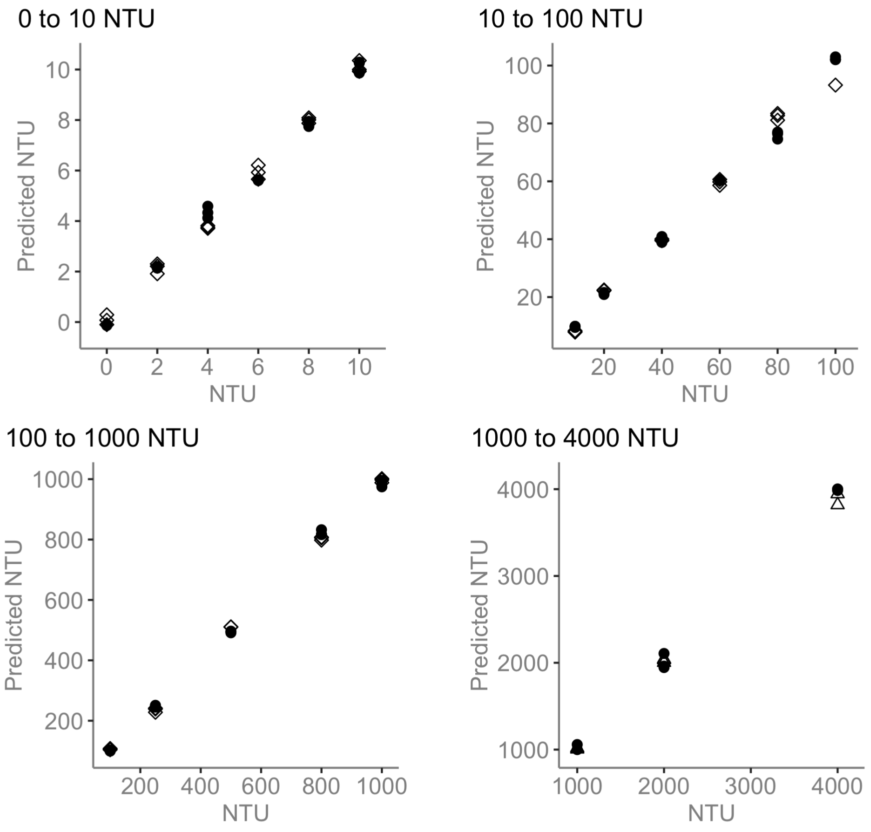

Figure S 8. Model predictions of NTU for Sensor 3 (black point) and Sensor 4 (white diamond) in four different ranges: TOP-LEFT 0-10 NTU; TOP-RIGHT 10-100 NTU; BOTTOM-LEFT 100-1000 NTU; and BOTTOM-RIGHT 1000-4000 NTU.

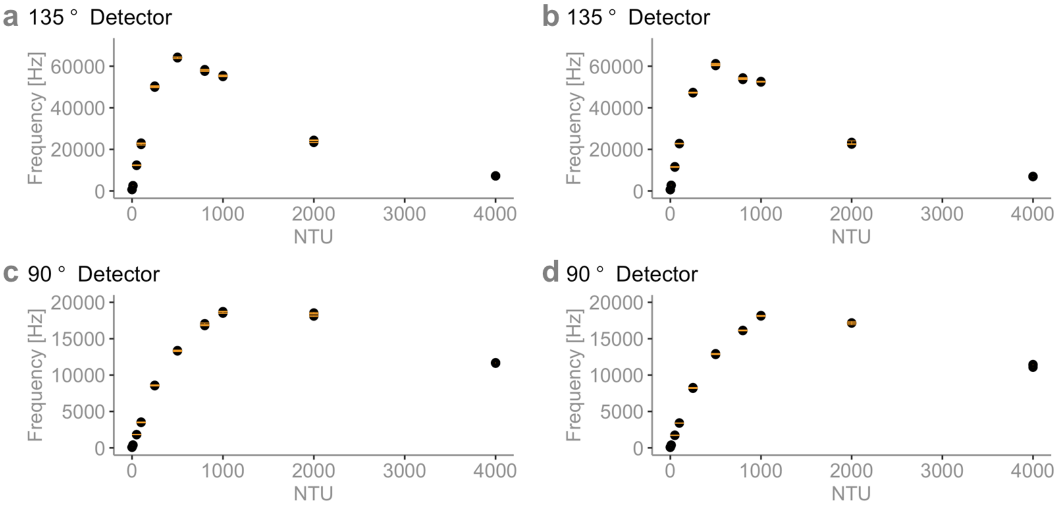

Figure S 9. Quantity of light in hertz [Hz] reaching the detector for each NTU dilution. The mean (black points) and errors (orange bars from +/- standard deviation) for Sensor 3 under ambient light without long-pass filters (a and c) and with long-pass filters (b and d). The mean and error were found by subtracting the mean of when the LED was off to the mean of when the LED was on.

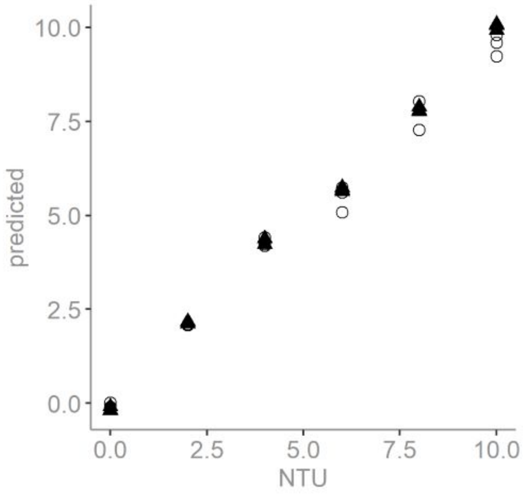

Figure S 10. Model predictions of NTU for Sensor 3 with (white circles) and without (black triangles) long-pass filters in the 0-10 NTU range.

Supplementary Table S4

List of sensor versions for the second prototype.

Sensor name	Sensor version	Detector Orientations	Method
1	A	90°, 135°	Machined PVC
2	A	90°, 135°	Machined PVC
3	A	90°, 135°	Machined PVC
4	B	45°, 135°	Machined PVC
5	B	45°, 135°	Machined PVC
6	B	45°, 135°	Machined PVC
7	C	90°, 135°	3D printed
8	C	90°, 135°	3D printed

Supplementary Figure S11

NTU vs. SSC comparison of three E&H sensors (first CUS51D in light-grey, second CUS51D in mid-grey, CUS52D in dark grey). a) Feldspar powder in the full measurement range 0-16 g/L, b) Feldspar powder in the 0-2 g/L range, c) Feldspar powder in the 0-0.5 g/L range, d) Fieschertal sediment in the full measurement range 0-16 g/L, e) Fieschertal sediment in the 0-2 g/L range, f) Fieschertal sediment in the 0-0.5 g/L range. Error bars represent +/- one standard deviation.

All of the E&H sensors are rated for 0-4000 NTU (see Methods section for more details on E&H sensors). However, on the E&H website they state that the CUS51Ds are used in applications with medium to high turbidities[7], whereas the CUS52D is used at every quality control point in drinking water production (from inlet to outlet), down to the lowest turbidities[8].

We would like to investigate how these sensors report NTU at various sediment concentrations and if we could use commercial sensors to build a distributed turbidity sensor network. Panel a) shows that the two CUS51Ds report similar NTU readings in feldspar sediment at higher SSCs, meaning that these two sensors give "reproducible" results. Therefore, at higher concentrations (in feldspar) these two sensors can be placed in a catchment and their turbidities compared. However, at SSCs below 2 g/L (panels b and c) both CUS51D sensors are not able to provide reproducible or sensible results, since (no clear relationship between NTU and SSC), perhaps since these sensors are meant for high turbidities. Observing the CUS52D sensor (dark grey) in panel a), we find that the NTUs are higher relative to the two CUS51Ds between 3-6 g/L, abruptly peak at 7.5 g/L and read 10,000 NTU (possibly an error or bubbles present), and after 7.5 g/L the readings plateau at 3000 NTU. Since the CUS52D is meant for drinking water applications, possibly it is unable to take measurements above 7.5 g/L. However, this sensor is also not able to give sensible results below 2 g/L (panels b and c), although this is stated by E&H. Overall, the measurements from the CUS51Ds and CUS52D are non-comparable, since readings differ highly at SSCs above 2 g/L and no clear relationship between NTU and SSC can be observed below 2 g/L for both sensors.

Comparing these results to the Fieschertal sediment results in panels d-f, we see again that the CUS51Ds are able to report reproducible results for the full range of data except below 2 g/L, where no meaningful relationship between NTU and SSC can be observed. Additionally, all three sensors inexplicably plateau between 5-15 g/L (possibly due to bubbles). In panels d-e, we find that the CUS52D (dark grey) reports better results between 1-5 g/L (NTU follows some trend with SSC), which is expected. However, this sensor reports unusable results below 0.25 g/L. In both cases, for feldspar and Fieschertal sediments, the CUS51Ds' results cannot be compared to the CUS52D's results, even if both sensors are from the same manufacturer and have been calibrated from 0-4000 NTU. Therefore, the NTU values reported by these sensors should not be compared but analyzed individually. None of the sensors are able to give sensible results for the entire 0-16 g/L range. It should be noted that the sensors were borrowed from scientists who use them in the field, so recalibration could lead to better results.

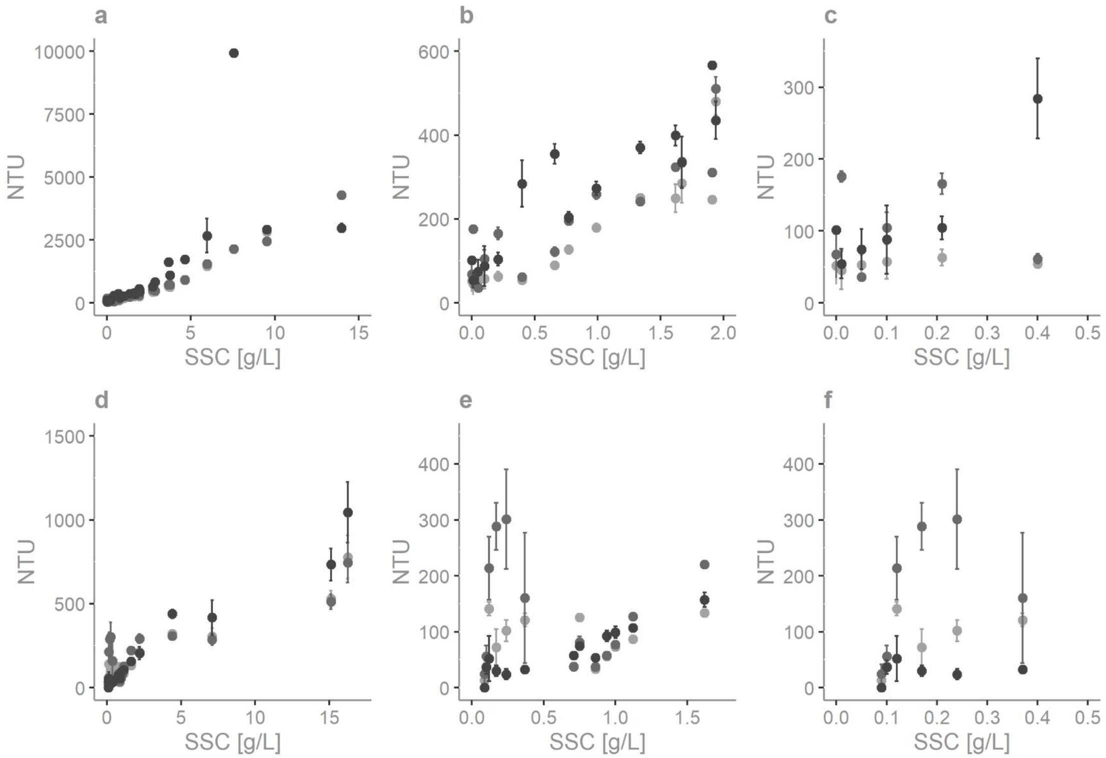

Supplementary Figure S12

Observed SSCs in Switzerland. The mean, 95% percentile, and maximum observed SSC in g/L at 13 stations of the Federal Office for the Environment (BAFU) network where automatic measurements are taking place. We consider these the typical Alpine river SSCs. There is an average 3,400 measurements at each station (twice weekly roughly). The mean across the 13 stations is 0.11 g/L. And the mean of 11 rivers, excluding the two rivers with the lowest mean SSC, is 0.129 g/L. The measurement that is exceeded on average 5% of the time is about 0.4 g/L. All of the maximum events fall below 23 g/L except for two events (at 55.6 g/L and 71.4 g/L).

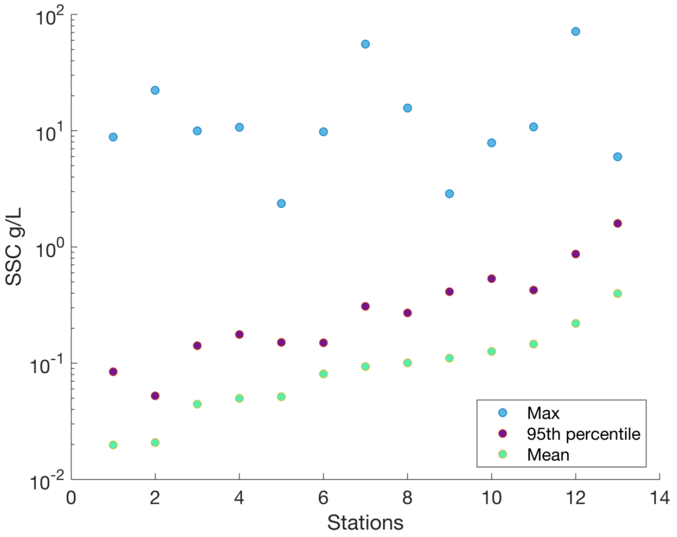

Supplementary Figure S13

Model predictions of NTU for Sensor 6 in four different ranges: TOP-LEFT 0-10 NTU; TOP-RIGHT 10-100 NTU; BOTTOM-LEFT 100-1000 NTU; and BOTTOM-RIGHT 1000-4000 NTU.

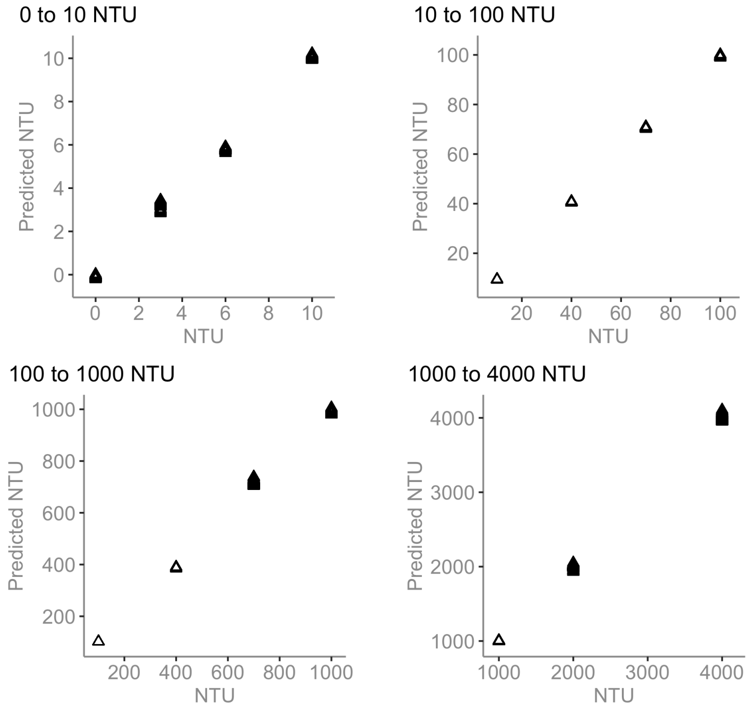

Supplementary Table S5

Model coefficients for sensor 6.

Sensor	Range [NTU]	α	β	γ	R^2	p-value
6	0-10	-10.62472490	0.01891566	-	0.9970	< 2.2e-16
6	10-100	-12.9745875	0.0204973	-	0.9996	< 2.2e-16
6	100-1000	1.039077e+2	-1.191820e-2	2.123267e-6	0.9983	< 2.2e-16
6	1000-4000	4.904038e+3	-3.846541e-1	9.306911e-6	0.9995	< 2.2e-16

Supplementary Discussion - Problems with calibrating NTU with Formazin

A sensor that is simply meant to provide a clarity estimate can undergo a calibration to NTU with formazin. This is the traditional calibration of a turbidity sensor and our open-sourced sensors can also be used and calibrated in this way. However, within this unit of measure is hidden many complex processes, such as particle size, configuration, refractive index, organic matter and other floating debris, algae, air bubbles and water discoloration (wavelength absorption), which all determine the spatial distribution of the scattered light intensity around a particle[6,9]. These processes cannot be deciphered from an NTU unit, especially when comparing units using sensors made by different manufacturers or by the same manufacturer but different sensor models. Kitchener[10] has shown that NTU is not a physically valid unit of turbidity (and suggests to record data in the SI unit of radiated intensity [mW/sr][11]) since it is based on the mass-concentrations of arbitrary polymer suspensions (e.g. Formazin), and not on the intrinsic optical properties of the particles in suspension. This is also what we see in Fig.5 (main manuscript). If the purpose of the sensor would be to determine what elements cause the water's clarity to change, then it might be better to calibrate one of our sensors directly to SSC and additionally equip it with other detectors to determine, for example, the presence of organic matter. This is what Matos[12] achieved when they equipped a 90° light scatter detector with a UV transmitter and receiver.

Another issue noted by Felix[13] is that during a flood event, commercial NTU-calibrated turbidimeters yielded up to 80% lower SSCs than those from Laser in-situ Scattering and Transmissiometry (LISST) and bottle samples when coarser particles were present. They mainly attribute this to the temporal variation in PSD; as coarser particles produce less attenuation and scattering at a given SSC (as we see with Fieschertal sediment in Fig.5 b and d (main manuscript)). For this reason, they recommend more advanced optical techniques for SSC such as LISST or Coriolis Flow- and Density Meter (CFDM) which were found to be less biased by temporary PSD variations than SSCs obtained by turbidimeters. However, these devices are generally unsuitable for field applications and raise the measurement costs to US$ 15 000[14] - 25 000[15] (for a CFDM) and between US$ 35 000 and 100 000 (for LISST[15]).

In their experiments, Felix[16] used a turbidimeter that was calibrated up to 4000NTU and they found that their NTU-SSC calibration was only valid for particles up to $d_{50} = 17\mu m$ (the variation in PSD isn't an issue when the particles present are relatively fine[14], which is the case during the majority of the time at the HPP Fieschertal storage tunnel). They found that the coarsest particles were transported according to a hysteresis pattern, affecting the NTU-SSC calibration when particles were coarser than $d_{50} = 17\mu m$, and they saw no clear correlation between SSC and d_{50}. However, they did find that the slope of their NTU-SSC calibration curve (eqn. 6[16]) decreases with coarser particles (as seen in our Fig.2 in the main manuscript). Nezu[17] and Parsons[18] found that SSC and PSD, for coarser particles such as sand, vary on the short term even in steady-state flow conditions due to the random nature of turbulence and coherent flow structures.

Therefore, PSD will affect our turbidity measurements since it is not measured by our sensors and so our sensors cannot overcome its influence. Perhaps by recognizing this issue and directly calibrating Hz-SSC, we can obtain a regression fit (Hz-SSC) which spans the entire particle size range; more accurately representing the "true" SSC and overcoming the limitation imposed by the manufacturer's standard Hz-NTU calibration. Additionally, by obtaining multiple samples during various flood events when larger particles are transported, we may improve the Hz-SSC relation since random errors due to temporal fluctuations in PSD are reduced by measuring at a suitably high frequency and time-averaging[16]. It would then be worth investigating whether our site-specific Hz-SSC calibration model can consistently capture SSC as both LISST and CFDM[14].

Supplementary Figure S14

Electronic schematic of second prototype. The sensor has five sub-circuits. A 9V battery is connected to the Power Supply Circuit, which supplies power to the entire system. The P-channel MOSFET here is used to prevent any damage to the ESP32 microchip in case the battery is accidentally connected backwards. The DC-DC converter is used to step down the battery voltage from 9V to the ESP32-WROOM-32D input voltage of 5V and a slide switch is installed between this converter and the 5V line to turn the sensor on and off.

Both switch circuits are controlled from pins on the ESP32-WROOM-32D module by setting the pins from high (3.3V) to low (0V). Both circuits have an N- and P-channel MOSFET combination which are used to supply or cut the current flowing from +5V. Switch 1 Circuit is used to turn off and on all of the electronic components (detectors, SD card reader) between readings to save power. By setting pin 27 on the ESP32-WROOM-32D to high and low in the code for Switch 1 Circuit, current would either flow from 5V to SWI_1 (high) or no current would flow (low). When current flows, everything connected to SWI_1 is supplied with power. The same principle is used for Switch 2 Circuit; when pin 19 on the ESP32-WROOM-32D is set to high, current flows from +5V to ground and supplies power to the TSHG6200 LED. When pin 19 is low, the LED is off.

The ESP is programmed to take a measurement every minute and between readings it will enter deep sleep. When the ESP is taking a measurement, it will set pin 27 to high and supply power to the SD Card Reader and the Detector Circuits. At the same time, it will take a frequency reading first with pin 19 set to low (LED off) and then again with pin 19 set to high (LED on), for the off-on differencing measurement. Once the differencing measurement is finished, the ESP will turn pin 27 low which should cut all power to the detectors and the SD card reader, then the ESP will enter deep-sleep[19] until it is time to take another measurement.

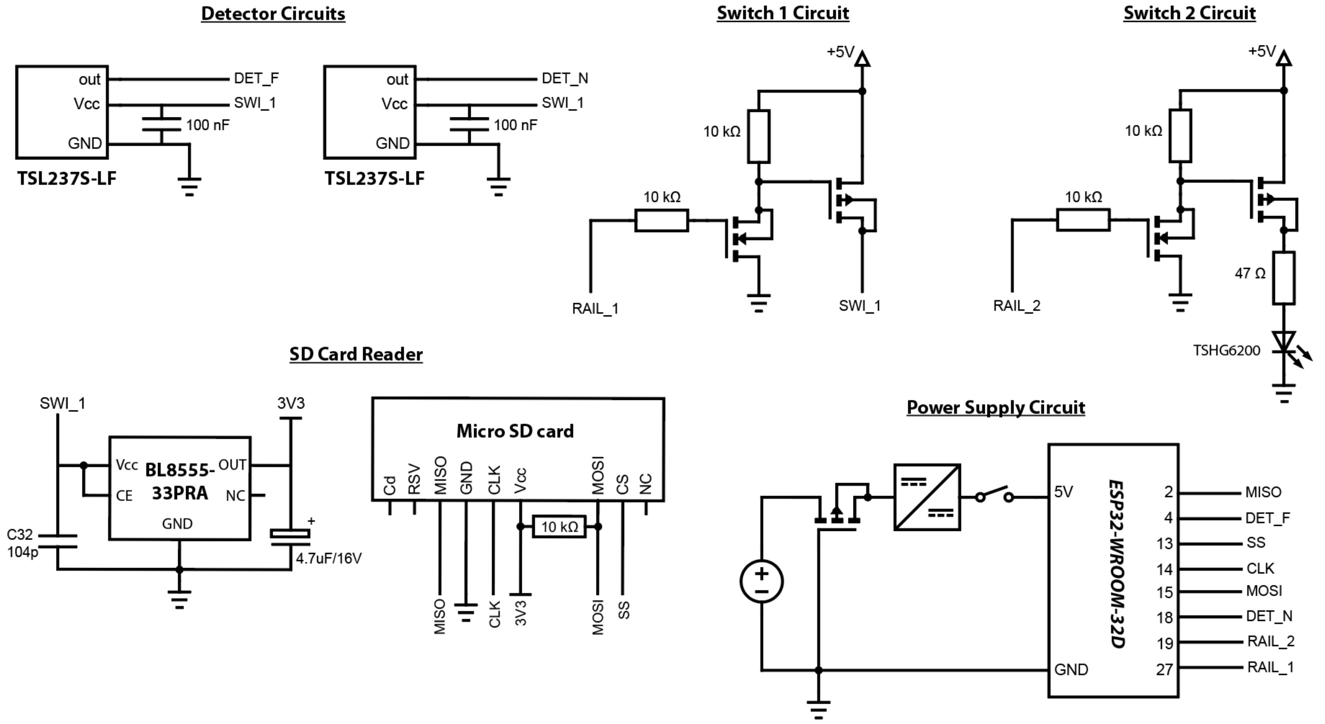

Supplementary Table S6

The cost of one open-source sensor (second prototype).

Item	Part number	Quantity	Cost/item [CHF]
ESP32 Dev board	ESP32-DEVKITC-32D		8.90
DC/DC Converter	TSR 1-2450E		3.53
MicroSD module	DFR0229		4.63
P-Channel MOSFET	AOI21357	3	0.58
N-Channel MOSFET	IRLZ44NPBF	2	0.96
Slide Switch	GF-123-0054		1.11
9V Battery			1.69
9V Leads			0.45
10k resistors	CFR50J10K	7	0.062
IR LED	TSHG6200		0.93
Detectors	TSL237-S-LF-ND	2	3.14
Capacitors	C315C104M5U5TA7303	2	0.09
Prototyping board	DFRobot FIT0099		1.34
Lenses	Jingliang Optical Technology Co.	2	2.24
O rings	O-ring NBR 70 36624 82x2,5mm	2	1.88
PVC sensing head	Machined by technician		20
Total			61.37

References

1. Droujko, J. Supporting make files for "open-source, low-cost, in-situ turbidity sensor for river network monitoring" (v1.0.0). DOI: 10.5281/zenodo.5789211 (2021).
2. Gillett, D. & Marchiori, A. A low-cost continuous turbidity monitor. *Sensors* **19**, 3039 (2019).
3. Felix, D. Experimental investigation on suspended sediment, hydro-abrasive erosion and efficiency reductions of croated pelton turbines. *VAW-Mitteilungen* **238** (2017).
4. Postolache, O. A., Girao, P. S., Pereira, J. D. & Ramos, H. M. G. Multibeam optical system and neural processing for turbidity measurement. *IEEE Sensors J.* **7**, 677–684 (2007).
5. Matos, T. *et al.* Design of a multipoint cost-effective optical instrument for continuous in-situ monitoring of turbidity and sediment. *Sensors* **20**, 3194 (2020).
6. Sadar, M. J. Turbidity science. technical information seriesbooklet no. 11. *Hach Co. Loveland CO* **7**, 8 (1998).
7. Endress + Hauser. Suspended solids sensor turbimax cus51d. https://www.ch.endress.com/en/field-instruments-overview/liquid-analysis-product-overview/suspended-solids-turbidity-digital-sensor-cus51d (2021). [Online; accessed 4-August-2021].
8. Endress + Hauser. Turbidity sensor turbimax cus52d. https://www.ch.endress.com/en/field-instruments-overview/liquid-analysis-product-overview/turbidity-drinking-water-sensor-cus52d (2021). [Online; accessed 4-August-2021].
9. Dabney, S., Locke, M. & Steinriede, R. Turbidity sensors track sediment concentrations in runoff from agricultural fields. In *Proceedings of the Eighth Federal Interagency Sedimentation Conference (8thFISC), Reno, NV, USA* (2006).
10. Kitchener, B. G., Wainwright, J. & Parsons, A. J. A review of the principles of turbidity measurement. *Prog. Phys. Geogr.* **41**, 620–642 (2017).
11. Kitchener, B. G. *et al.* A low-cost bench-top research device for turbidity measurement by radially distributed illumination intensity sensing at multiple wavelengths. *HardwareX* **5**, e00052 (2019).
12. Matos, T. *et al.* Development of a cost-effective optical sensor for continuous monitoring of turbidity and suspended particulate matter in marine environment. *Sensors* **19**, 4439 (2019).
13. Felix, D., Albayrak, I. & Boes, R. M. Field measurements of suspended sediment using several methods. In *E-Proceedings of the 36th IAHR World Congress, 28 June-3 July, 2015, The Hague, the Netherlands* (IAHR, 2015).

14. Felix, D., Albayrak, I. & Boes, R. M. Continuous measurement of suspended sediment concentration: Discussion of four techniques. *Measurement* **89**, 44–47 (2016).

15. Rai, A. K. & Kumar, A. Continuous measurement of suspended sediment concentration: Technological advancement and future outlook. *Measurement* **76**, 209–227 (2015).

16. Felix, D., Albayrak, I. & Boes, R. M. In-situ investigation on real-time suspended sediment measurement techniques: Turbidimetry, acoustic attenuation, laser diffraction (lisst) and vibrating tube densimetry. *Int. journal sediment research* **33**, 3–17 (2018).

17. Nezu, I. & Nakagawa, H. *Turbulence in open-channel flows* (Routledge, 2017).

18. Parsons, A. J., Cooper, J. & Wainwright, J. What is suspended sediment? *Earth Surf. Process. Landforms* **40**, 1417–1420 (2015).

19. ESP-IDF Programming Guide. Sleep modes. https://docs.espressif.com/projects/esp-idf/en/latest/esp32/api-reference/system/sleep_modes.html (2021). [Online; accessed 6-October-2021].
